# Immune infiltration-related genes regulate the progression of AML by invading the bone marrow microenvironment

**DOI:** 10.3389/fimmu.2024.1409945

**Published:** 2024-07-12

**Authors:** Shuangmei Yu, Jiquan Jiang

**Affiliations:** ^1^ Department of Radio-immunity, Heilongjiang Provincial Hospital, Harbin, China; ^2^ Department of Laboratory Diagnosis, The Second Affiliated Hospital of Harbin Medical University, Harbin, China

**Keywords:** acute myeloid leukemia, immune infiltration-related genes (IIRGs), WGCNA, LASSO, immune infiltration analysis, bone marrow microenvironment

## Abstract

In this study, we try to find the pathogenic role of immune-related genes in the bone marrow microenvironment of AML. Through WGCNA, seven modules were obtained, among which the turquoise module containing 1793 genes was highly correlated with the immune infiltration score. By unsupervised clustering, the turquoise module was divided into two clusters: the intersection of clinically significant genes in the TCGA and DEGs to obtain 178 genes for mutation analysis, followed by obtaining 17 genes with high mutation frequency. Subsequently, these 17 genes were subjected to LASSO regression analysis to construct a riskscore model of 8 hub genes. The TIMER database, ImmuCellAI portal website, and ssGSEA elucidate that the hub genes and risk scores are closely related to immune cell infiltration into the bone marrow microenvironment. In addition, we also validated the relative expression levels of hub genes using the TCGA database and GSE114868, and additional expression levels of hub genes in AML cell lines *in vitro*. Therefore, we constructed an immune infiltration-related gene model that identify 8 hub genes with good risk stratification and predictive prognosis for AML.

## 1 Introduction

Acute myeloid leukemia is a group of hematological malignancies with multiple genetic characteristics and high clinical heterogeneity (1, 2). Although intensive chemotherapy, novel targeted drug therapy, and allogeneic stem cell transplantation have made significant progress and have high efficacy (3). However, the prognosis of patients with AML is still poor, with high relapse and mortality rates (4). It has been reported that the estimated 5-year survival rate of AML patients with good cytogenetics is approximately 55%. In comparison, the estimated 5-year survival rates of patients with intermediate and poor cytogenetics are only 24% and 5%, respectively (5). Therefore, it is crucial to determine patient risk stratification and prognostic assessment during diagnosis.

Currently, the generally recognized clinically relevant prognostic parameters of AML, such as age, white blood cell count, and cytogenetic changes, provide some clues for prognosis prediction and risk stratification assessment, but the results are unsatisfactory (6). Notably, in recent years, the rapid development of high-throughput sequencing and chip detection technologies has provided essential insights into the pathology and pathogenesis of various diseases (7). In addition, bioinformatics analysis is widely used in tumor research and prognosis prediction, and analyzing public databases and mining novel prognostic markers provides a broader perspective for the clinical diagnosis and prognosis assessment of AML (8). In particular, the Weighted Gene Co-expression Network Analysis (WGCNA) (9), as a comprehensive biological algorithm, has significant advantages in analyzing association patterns between genes. Module clustering of genes with similar expression patterns and correlation analysis between modules and clinical traits are two highlights of WGCNA.

Actually, the relationship between the immune system and tumors is intricate and can form a complex network called the tumor microenvironment (TME) (10, 11). In the TME, tumor cells communicate not only with each other but also with stromal cells and immune cells (12). The constant interaction between tumor cells and the tumor microenvironment plays a decisive role in tumor initiation, progression, metastasis, response to treatment, and predicting prognosis (13). Similarly, the occurrence and development of AML are closely related to the bone marrow microenvironment (14). It has been reported that early resistance is mediated by the bone marrow microenvironment, which protects residual leukemia cells and impacts on chemotherapy efficacy over time (15). However, research on how immune cells infiltrate AML and promote AML progression remains to be further elucidated.

In this study, we aimed to decipher the pathogenic role of immune infiltration-related genes in the bone marrow microenvironment in AML. First, we obtained immune infiltration-related genes through WGCNA, unsupervised clustering analysis and GSCA mutation analysis. Subsequently, a riskscore model was established through LASSO COX regression analysis. More importantly, we conducted an in-depth analysis of the immune-infiltrated tumor microenvironment on the gene signature established in this study. In addition, the relative expression levels of immune infiltration-related genes in the riskscore signature were verified in the external dataset and AML cell lines. Our study combines WGCNA, GSCA mutation analysis, and immune infiltration tumor microenvironment analysis to reveal the complexity of AML further. It provides a new perspective for clinical risk stratification and prediction of prognosis, which is helpful for elucidating and exploring the pathogenesis of AML.

## 2 Materials and methods

### 2.1 Data acquisition and processing

Bone marrow RNA sequencing data and corresponding clinical information of 151 AML patients were retrieved from TCGA-LAML dataset which is the subset of The Cancer Genome Atlas (TCGA, https://portal.gdc.cancer.gov/) database. Additionally, we obtained the transcriptomic profiles of GSE71014, which consists of 104 AML samples, and GSE114868, which consists of 194 AML samples and 20 normal samples from the Gene Expression Omnibus (GEO, http://www.ncbi.nlm.nih.gov/geo) database (16) (The information was listed in **Supplementary Table S1**). The datasets were utilized as the validation set.

### 2.2 Weighted gene co-expression network analysis

The Weighted Gene Co-expression Network Analysis (WGCNA) is an algorithm used to identify gene co-expression networks by high-throughput expression profiles of mRNAs with different traits. Here, immune cell infiltration-related genes were identified using the “WGCNA” R package. The correlation between gene expression and sample trait (immune cell infiltration score) was determined by the criterion of gene significance (GS) > 0.9 and module membership (MM) > 0.9.

### 2.3 Unsupervised clustering analysis and identification of differentially expressed genes

The R package “ConsensusClusterPlus” presented the unsupervised subgroups and clusters of high immune-related modular genes identified by WGCNA. We used the “limma” R package to identify differentially expressed genes (DEGs) in the subgroups generated by unsupervised clusters. The rigorous filtering threshold is |log2 fold-change (FC)| > 1.0, and the false discovery rate (FDR)< 0.05. The DEGs were then visualized with a volcano plot and heatmap.

### 2.4 GO, KEGG, GSEA and GSVA enrichment analyses

Gene Ontology (GO) and Kyoto Encyclopedia of Genes and Genomes (KEGG) pathway enrichment analyses were performed using the “clusterProfiler” package. Furthermore, the GSEA (version 4.1.0) (17) software was utilized to analyze gene function concerning high- and low-risk scores, the rigorous threshold is adjust p value< 0.05 and FDR< 0.25. Similarly, GSVA enrichment analysis revealed the disparities in GSVA scores between the high-risk and low-risk samples (18).

### 2.5 Mutation analysis of GSCA

The Gene Set Cancer Analysis (GSCA, http://bioinfo.life.hust.edu.cn/GSCA/#/) is a web-based database that offers an interactive and comprehensive platform (19). The GSCA database includes a “mutation” module that allows for the analysis of mutation frequencies in specific genes of interest, mainly single nucleotide variants.

### 2.6 Univariate and multivariate COX and LASSO regression analysis

We employed the least absolute shrinkage and selection operator (LASSO) method to construct a refined and simplified COX model for predicting patient risk and prognosis to mitigate the risk of overfitting prognostic risk models. The risk score (RS) formula is defined as follows:


$$RS=\sum_{i=1}^{n}Coef_{i}\;\times \;Exp_{i}$$

Among them, *n* represents the number of genes included in the prognostic signature, *Coefi* represents the LASSO coefficient of gene *i*, and *Expi* indicates the expression value of gene *i*.

The model was used to calculate riskscores based on the expression of the related genes in different AML samples. Additionally, GSE71014 was utilized to compute the risk score for each patient. Nomogram was constructed to visualize the results of multiple factor regression analysis by “RMS” R software package.

### 2.7 Immune infiltration analysis

In order to assess the abundance of immune cell infiltration, a series of analyses about immune cell infiltration were conducted in AML. The single-sample Gene Set Enrichment Analysis (ssGSEA) method, available in the “GSVA” R package, was employed to assess the infiltration level of immune cells between high-risk and low-risk groups. We employed Immune Cell Abundance Identifier (ImmuCellAI, http://bioinfo.life.hust.edu.cn/web/ImmuCellAI/) to identify the levels of immune cell infiltration in both high-risk and low-risk groups. Specifically, we utilized the TIMER2.0 database (20) (https://timer.cistrome.org/) to analyze the differences in infiltration between mutant and wild-type genes involved in the LASSO model across different immune cell types.

### 2.8 Validation of mRNAs relative expression level

We selected the TCGA-GTEx dataset and GSE114868 dataset to verify the differential expression of hub genes in AML and normal tissues. In addition, we validated the relative expression levels of hub genes in cell lines.

Human AML cells (HL-60 and THP-1) were purchased from Procell (Wuhan, China), and human AML cells (MOLM-13) were purchased from MeisenCTCC (Jinhua, China), respectively. AML cells and normal cells (bone marrow mesenchymal stem cells [BMSC]) were cultured in RPMI 1640 (Sigma) and DMEM/F12 (gibco), respectively, with 10% fetal bovine serum (gibco). Total RNA was extracted from BMSC and AML cell lines using the TRIzol reagent (Invitrogen). RT-qPCR assays were performed using the SYBR Green Realtime PCR Master Mix (TOYOBO). The mRNA expression levels were normalized to GADPH, and each sample was tested in triplicate. The primer sequences of mRNAs and GAPDH were synthesized by BGI (Beijing, China). The primers used in this study are listed in **Supplementary Table S2**.

### 2.9 Statistical analysis

All analyses were performed using R v. 4.2.1 (https://www.R-project.org). Data were presented as means ± standard error of the mean (SEM) in at least three independent experiments and analyzed with GraphPad Prism 9.3.1. The Wilcoxon rank sum test and the Welch’s t-test were used to compare the expression differences of unpaired samples between the two groups. The Wilcoxon rank sum test was utilized to analyze the relations between the clinicopathological features and risk score.

The detailed flowchart is exhibited in **Supplementary Figure S1**.

## 3 Results

### 3.1 The identification of modules related to immune infiltration through WGCNA

**Figure 1:**
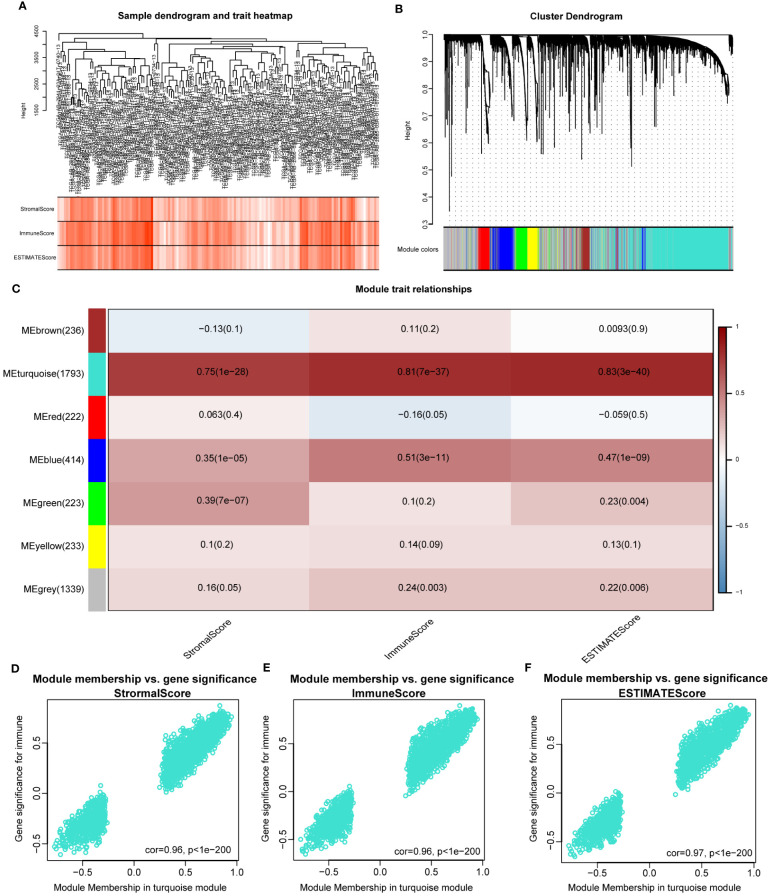
Identifying immune-related modules by WGCNA. **(A)**. The cluster was based on the transcriptome data from TCGA-LAML. The color intensity represents the immune factor (StromalScore, ImmuneScore and ESTIMATEScore). **(B)**. Hierarchical cluster analysis was performed to detect co-expression modules with corresponding colors. Each branch of the tree diagram represents genes, and genes clustered into the same module are assigned the same module color. **(C)**. Module-trait heatmap revealing the relationship between modules and immune factors, including StromalScore, ImmuneScore and ESTIMATEScore. The red refers to a positive correlation, while the blue indicates a negative correlation. **(D–F)**. The correlation analysis between membership (MM) in turquoise module and gene significance (GS) for immune factor.

The WGCNA algorithm was used to identify modules related to immune infiltration (including StromalScore, ImmuneScore, and ESTIMATEScore). When the scale-free topology fitting index reached 0.9, the soft-thresholding power β was 7 (**Supplementary Figure S2A**). Seven modules were identified under the parameter settings of minModuleSize=100 and mergeCutHeight=0.15 (**Figures 1A, B**). According to the correlation coefficient and P-value, the MEturquoise module was the module with the strongest correlation with scores (StromalScore, r = 0.75, P = 1e-28; ImmunoScore, r = 0.81, P = 7e-37; ESTIMATEScore, r = 0.83, P = 3e-40; **Figure 1C**). In addition, GS and MM in the turquoise module were highly correlated, indicating that this module was most significantly correlated with immune infiltration (**Figures 1D–F**). For this reason, we selected the MEturquoise module containing 1793 genes as the key module for subsequent analysis.

In order to investigate the potential mechanism of co-expressed genes in the turquoise module, we conducted GO and KEGG enrichment analysis. The results indicated that these genes were highly enriched in terms of “positive regulation of cytokine production” (**Supplementary Figure S2B**), “external side of plasma membrane” (**Supplementary Figure S2C**), “phonological binding” (**Supplementary Figure S2D**), “Transcriptional misregulation in cancer” (**Supplementary Figure S2E**). The details of the results are shown in **Supplementary Table S3**. Overall, the genes in the turquoise module were highly correlated with the immunological biological processes of AML.

### 3.2 Unsupervised clustering analysis of turquoise module

**Figure 2:**
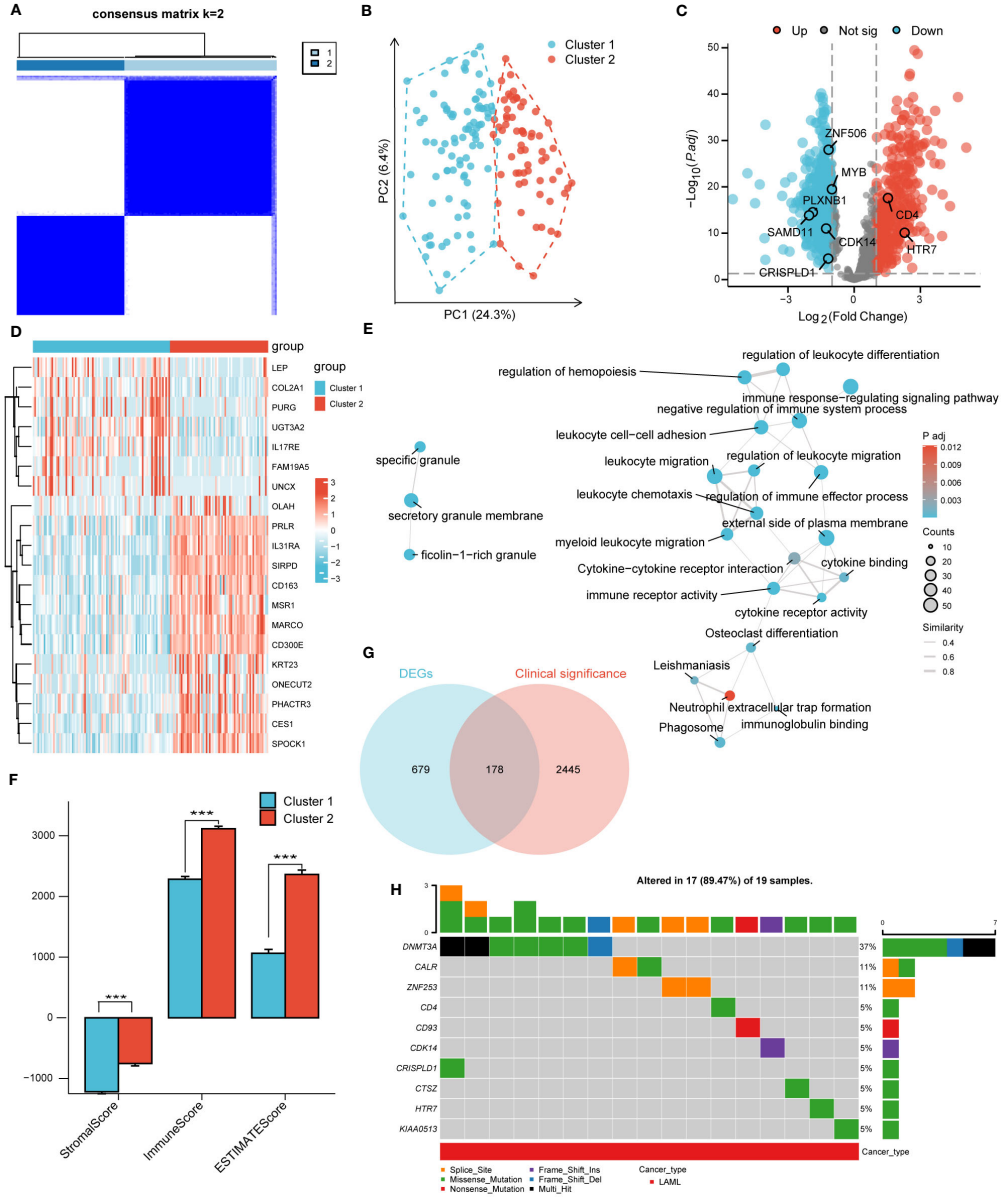
Unsupervised clustering analysis. **(A)**. Consensus clustering matrix of turquoise module for k=2. **(B)**. The principal component analysis (PCA) shows a different distribution of the two clusters. **(C)**. Volcano plot of DEGs between cluster 1 and cluster 2. |log2FoldChange| > 1 and adj. P< 0.01 were identified as significant DEGs. The red dots represent up-regulated genes and the blue dots represent down-regulated genes. **(D)**. Heatmap of DEGs between cluster 1 and cluster 2. **(E)**. Functional enrichment analysis of DEGs between cluster 1 and cluster 2. **(F)**. Immune Scores of ESTIMATE algorithm between cluster 1 and cluster 2. **(G)**. Intersection of DEGs and clinical significance genes by Venn. **(H)**. The top 10 mutated genes and variant classification in 178 candidate genes. (*** P<0.001).

According to the corresponding cumulative distribution function and K value function δ area, when K = 2, the curve exhibited stable aggregation (**Supplementary Figures S3A, B**). Therefore, the turquoise module was divided into two subgroups (Cluster 1: 88 samples; Cluster 2: 63 samples; **Figure 2A**). The principal component analysis (PCA) clearly distinguished the two clusters (**Figure 2B**). The results were also validated using t-distributed Stochastic Neighbor Embedding (t-SNE) and Uniform Manifold Approximation and Projection (UMAP) analysis (**Supplementary Figures S3C, D**).

Next, differential gene expression analysis was performed on clusters 1 and 2, with 857 DEGs generated, including 352 significantly upregulated genes and 505 significantly downregulated genes. The DEGs were displayed through volcano plots and heatmaps (**Figures 2C, D**). We conducted GO and KEGG analyses to better understand the biological processes and signaling pathways related to DEGs (**Figure 2E**). The GO and KEGG analysis results showed that DEGs were mainly enriched in biological processes such as “myeloid Leukocyte migration”, “negative regulation of immune system process” and in signaling pathways such as “Phagosome”, “Cytokine-cytokine receptor interaction”. In addition, the StromalScore, ImmuneScore, and ESTIMATEScore evaluated by the ESTIMATE algorithm showed that the scores of cluster 2 were significantly higher than that of cluster 1 (**Figure 2F**).

### 3.3 Screening of candidate genes and GSCA mutation analysis

A univariate COX regression analysis was conducted to identify 2623 clinically significant genes in the TCGA-LAML dataset, with a strict screening criterion of adj P<0.01. The Venn diagram was used to intersect DEGs and clinically significant genes, generating 178 candidate genes (**Figure 2G**). The pathogenesis of AML was closely related to genetic abnormalities and gene mutations. Therefore, we conducted mutation analysis on 178 candidate genes through the GSVA website and obtained 17 genes with high mutation frequencies. The 17 genes were *CALR*, *CD4*, *CD93*, *CDH23*, *CDK14*, *CRISPLD1*, *CTSZ*, *DNMT3A*, *HTR7*, *KIAA0513*, *LILRB1*, *LRP1B*, *MYB*, *PLXNB1*, *SAMD11*, *ZNF253*, *ZNF506*. Here, we only presented the top 10 mutated genes (**Figure 2H**; **Supplementary Figure S4A**). The mutation categories and variant types were shown in **Supplementary Figures S4B–F**.

### 3.4 Construction and validation of the prognostic model based on eight hub genes

**Figure 3:**
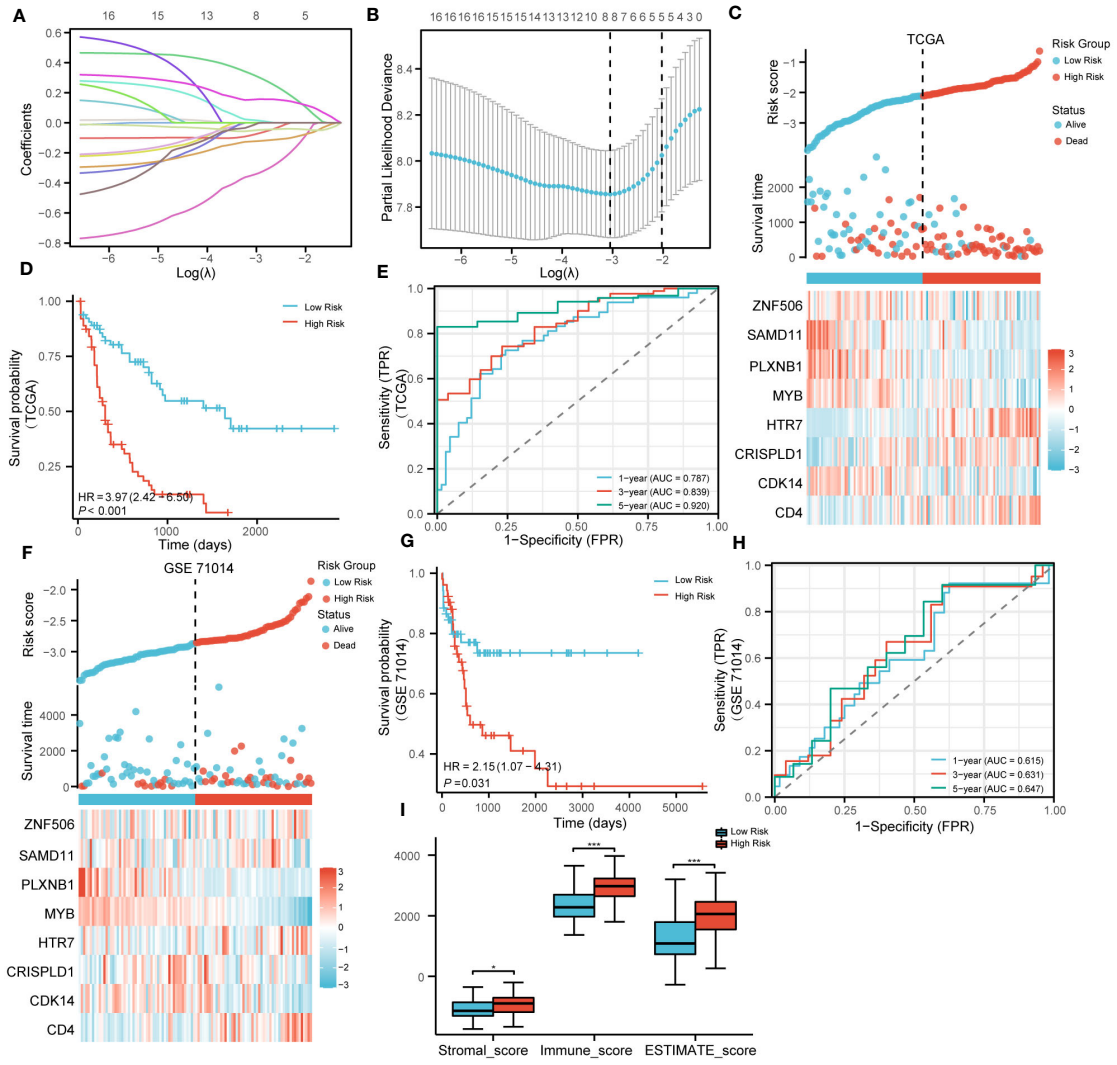
Construction and validation of prognosis model. **(A)**. LASSO coefficient profiles of 17 genes with high mutation frequency. **(B)**. Partial likelihood deviation of LASSO coefficient distribution. **(C)**. The survival status of patients and the expression of genes with different risk grades in the TCGA cohort. **(D)**. Kaplan–Meier survival analysis based on risk score in the TCGA cohort. **(E)**. The predictive capacity of the risk score for the 1- year, 3-year and 5- year survival rates. **(F–H)**. Stratified survival analysis of risk models and clinical characteristics in external GSE71014. **(I)**. Scores of ESTIMATE algorithm between low riskscore and high riskscore. (* P<0.05; ***P<0.001).

The LASSO algorithm was used to further screen 17 candidate genes with high mutation frequency for prognostic model construction. Eight characteristic genes were identified through the lowest cross-validation error: *ZNF506*, *SAMD11*, *PLXNB1*, *MYB*, *HTR7*, *CDK14*, *CRISPLD1* and *CD4* (**Figures 3A, B**). The formula of riskscore is: riskscore = gene expression × coefficient (**Table 1**). Patients were divided into high-risk and low-risk groups according to the median riskscore as the cutoff value (cut off = -2.10281) (**Figure 3C**). Kaplan-Meier survival analysis showed that the OS of high-risk group patients was significantly lower than that of low-risk group patients (**Figure 3D**).

**Table 1:** The genes and coefficient in LASSO model.

Gene Symbol	Description	coefficient
*CD4*	CD4 Molecule	0.0175
*CDK14*	Cyclin Dependent Kinase 14	-0.0782
*CRISPLD1*	Cysteine Rich Secretory Protein LCCL Domain Containing 1	0.3169
*HTR7*	5-Hydroxytryptamine Receptor 7	0.1547
*MYB*	MYB Proto-Oncogene, Transcription Factor	-0.3450
*PLXNB1*	Plexin B1	-0.1233
*SAMD11*	Sterile Alpha Motif Domain Containing 11	-0.0508
*ZNF506*	Zinc Finger Protein 506	-0.0135

Strikingly, Kaplan-Meier survival analysis was performed on the eight hub genes, and the results showed that these genes have a high predictive ability for AML (**Supplementary Figures S5A–H**). Time-dependent ROC curves were calculated, and the AUC values for 1, 3, and 5 years were all greater than 0.75, indicating that the risk model has good performance (**Figure 3E**). In addition, we also verified the effectiveness of the risk model on GSE71014. It was encouraging that the survival status and gene expression were consistent with those in the TCGA cohort, and excellent prognostic ability was also demonstrated (**Figures 3F–H**). In summary, the prognostic model we constructed demonstrated excellent predictive performance. In addition, compared with the low-risk group, the Stromalscore, Immunescore, and ESTIMATEscore in the high-risk group were significantly increased (**Figure 3I**).

### 3.5 Correlation analysis between riskscore and clinical features

In order to clarify the correlation between riskscores and clinical features, the riskscores of patients were compared based on the clinical characteristics of different groups. In the high-risk group, the proportion of age > 60, chromosomal abnormalities, and NPMc mutations significantly increased (**Supplementary Figures 6A–P**).

### 3.6 Screening of independent prognostic factors and construction of nomogram

**Figure 4:**
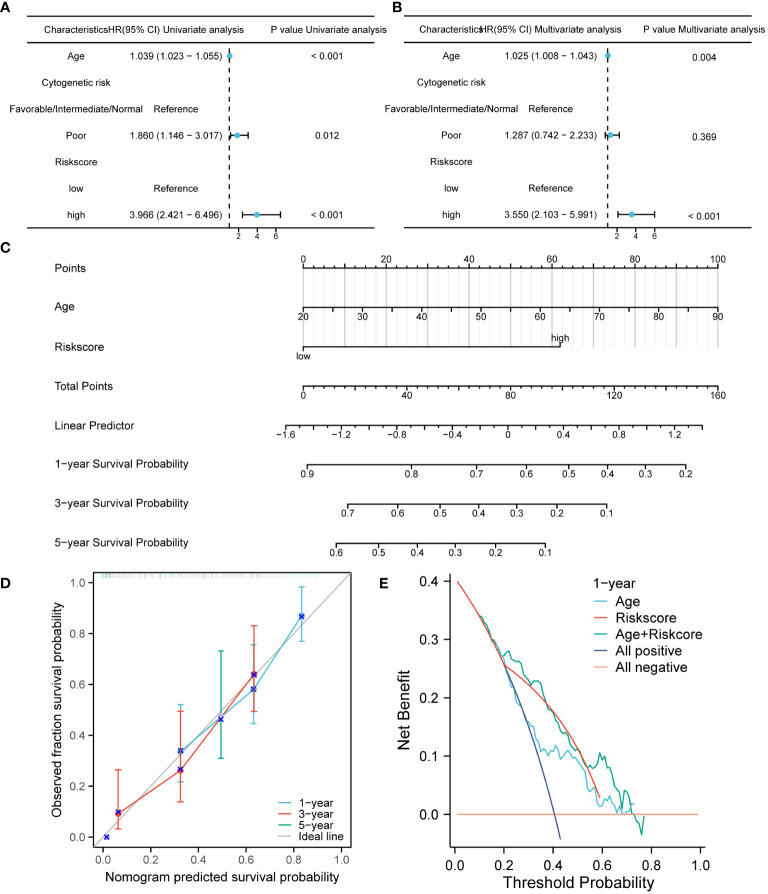
The construction and verification of the nomogram model for survival prediction. **(A, B)**. Univariate and multivariate COX regression analysis of riskscore and clinical characteristics. **(C)** The nomogram constructed by combining riskscore and age. **(D)**. The calibration curve of the nomogram. **(E)**. The DCA of the nomogram.

The prognostic performance of the risk model was obtained by conducting univariate and multivariate COX regression analysis with riskscore and a variety of clinical features (age and Cytogenetic risk). The univariate COX regression analysis of the TCGA cohort showed that age, riskscore, and Cytogenetic risk were risk factors for AML (**Figure 4A**). The results of multivariate COX analysis indicated that age and riskscore were independent prognostic factors for AML patients (**Figure 4B**). Then, we combined age and riskscore to establish the nomogram for survival prediction (C-index=0.736; **Figure 4C**). The prediction results of 1-year, 3-year, and 5-year OS indicated that the survival rate predicted by nomogram closely matches the best predictive performance (**Figure 4D**). Decision curve analysis (DCA) also demonstrated the clinical application value of constructing the nomogram (**Figure 4E**).

### 3.7 GSEA and GSVA between high-risk and low-risk groups

**Figure 5:**
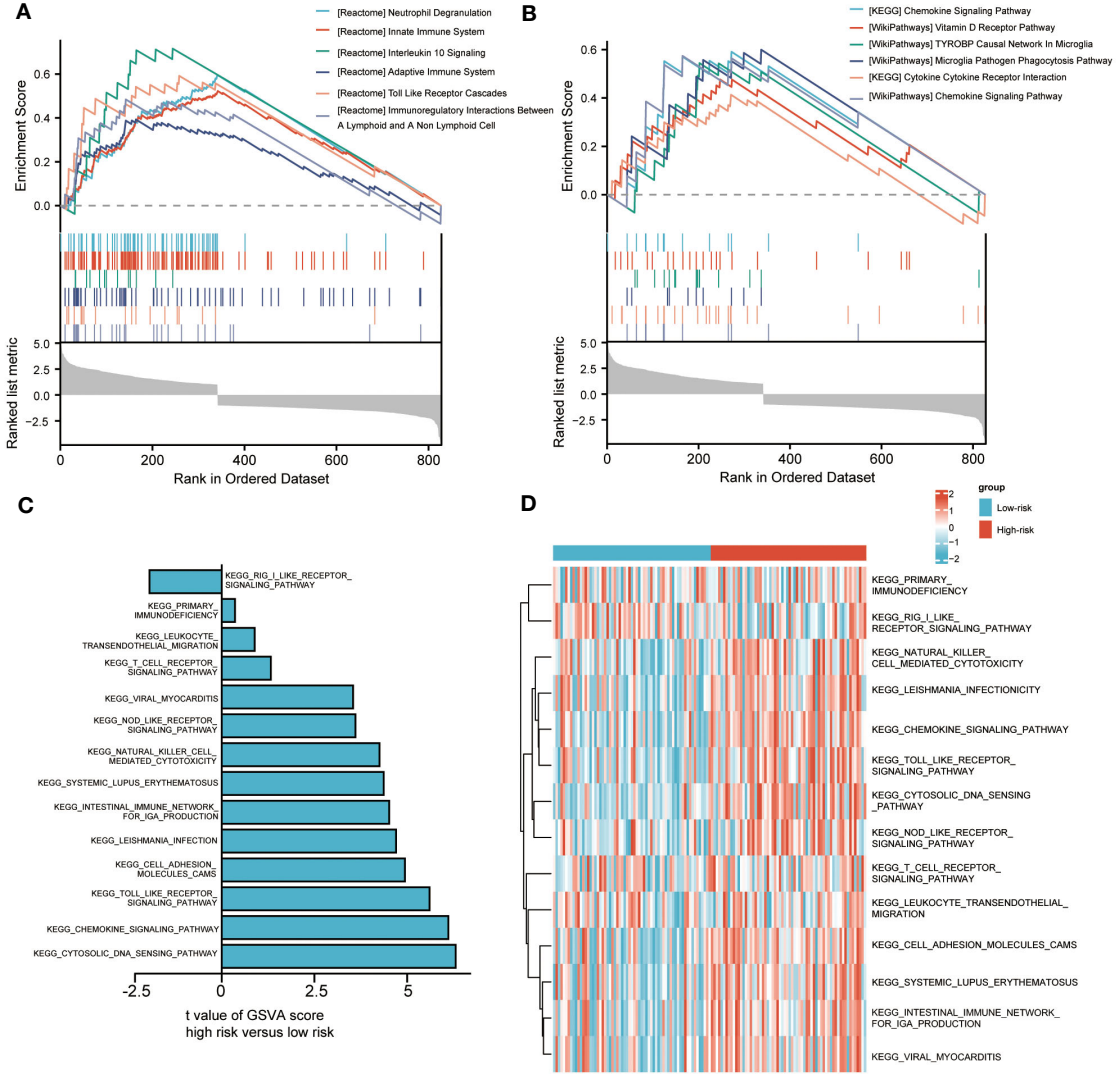
Biological characteristics between high-and low-risk groups. **(A, B)**. Gene set enrichment analysis (GSEA) of riskscore. **(C, D)**. Gene set variation analysis (GSVA) of riskscore.

To analyze the impact of high-risk and low-risk groups on AML progression, we conducted GSEA to determine the most significant enrichment pathway between the two risk groups. The results showed that the high-risk group was significantly enriched in processes such as “Innate Immune System”, “Adaptive Immune System” and “Toll-Like Receptor Cascades” (**Figures 5A, B**, **Supplementary Table S5**).

We also used GSVA to explore the differences in biological behavior between high-risk and low-risk groups. Compared with the low-risk group, the high-risk group was significantly enriched in biological pathways, such as “PRIMARY IMMUNODEFICIENCY”, “LEUKOCYTE TRANSENDOTHELIAL MIGRATION” and “NOD LIKE RECEPTOR SIGNALING PATHWAY”. On the contrary, compared with the high-risk group, the low-risk group only enriched with “RIG I LIKE RECEPTOR SIGNALING PATHWAY” (**Figures 5C, D**). The above biological processes were closely related to the immune system and immune cells.

### 3.8 Immune microenvironment landscape of AML

In order to better elucidate the immune microenvironment of AML, we conducted a series of immune infiltration tumor microenvironment analyses using various algorithms.

**Figure 6:**
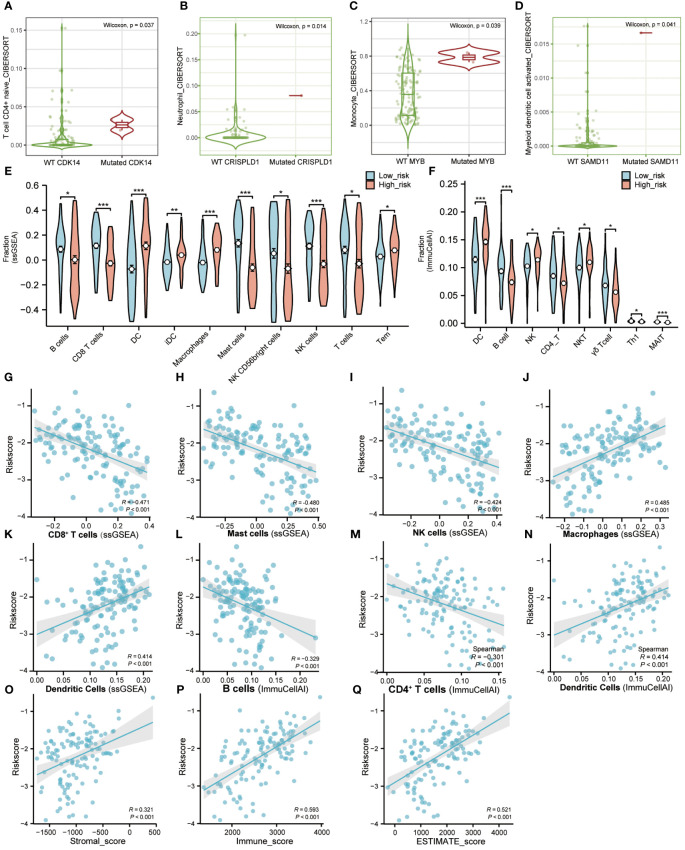
Immune microenvironment landscape of AML. **(A–D)**. TIMER database was used to validate the relationship between the mutation of genes and the infiltration level of immune cells in AML. **(E)**. The proportion of immune infiltration between high-risk and low-risk groups using “GSVA” R package. Red represents the high-risk group and blue represents the low-risk group. **(F)**. The difference of immune infiltration between high-risk and low-risk groups in the “ImmuCellAI” algorithm. Red represents the high-risk group and blue represents the low-risk group. **(G–N)**. The correlation analysis between immune cells and riskscore. **(O–Q)**. The correlation analysis between immune factors and riskscore. (Tem, T effector memory cells; MAIT, Mucosal-associated invariant T cells). (* P<0.05; ** P<0.01; *** P<0.001).

We revealed the immune cell infiltration levels of eight hub genes mutations using several algorithms by the TIMER database. When using the CIRBERSORT deconvolution method, there was difference in immune cell infiltration during mutation of *CDK14*, *CRISPLD1*, *MYB*, and *SAMD11*, while there was no difference in *CD4*, *HTR7*, *PLXNB1*, and *ZNF506* (**Figures 6A–D**). In addition, we also applied other algorithms from the TIMER database to evaluate the level of immune cell infiltration between gene wild type and mutation, showed the same results above (**Supplementary Figure S7**).

ssGSEA was used to calculate the infiltration fraction of 20 immune cells in different risk groups. The results showed significant differences in the infiltration levels of 10 types of immune cells in the high-risk and low-risk groups (**Figure 6E**; **Figures S8A, C**). Correlation analysis also showed a positive correlation between riskscore and infiltration of Dendritic cells and Macrophage cells, while a negative correlation between riskscore and infiltration of CD8+T cells, Mast cells, and NK cells (**Figures 6G–K**).

Furthermore, we applied the ImmuCellAI portal to evaluate the infiltration abundance of 24 immune cells in different risk groups. Compared with the low-risk group, the infiltration proportion of DCs (p< 0.001), NK cells (p< 0.05), and NK-T cells (p< 0.05) were significantly increased in the high-risk group. On the contrary, the infiltration proportions of B cells (p< 0.001), CD4-T cells (p< 0.05), Gamma-delta cells (p< 0.05), Th1 cells (p< 0.05), and MAIT cells (p< 0.001) were significantly reduced (**Figure 6F**; **Figures S8B, D**). The correlation analysis of ImmuCellAI also showed a positive correlation between risk score with infiltration of Dendritic Cells and a negative correlation with infiltration of B cells and CD4+T cells (**Figures 6L–N**).

In addition, correlation analysis of riskscore and immune infiltration scores calculated by the ESTIMATE algorithm demonstrated a positive correlation between riskscore with Stromalscore, Immunescore, and ESTIMATEScore, respectively (**Figures 6O–Q**). The above results indicated that the LASSO model we constructed was closely related to immune infiltration.

### 3.9 Verification of the expression level of hub genes

**Figure 7:**
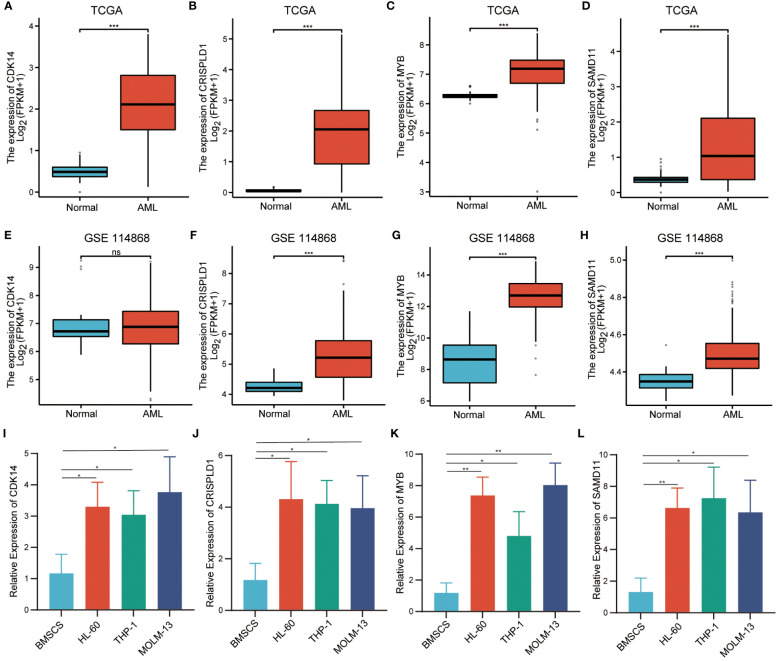
Verification of relative expression levels of hub genes. **(A–D)**. The relative expression levels of hub genes in TCGA cohort. **(E–H)**. The relative expression levels of hub genes in external validation dataset GSE114868. **(I–L)**. The relative expression levels of hub genes in BMSCs and AML cell. (*** P<0.001; no significance, ns).

It was found that only *CDK14*, *CRISPLD1*, *MYB*, and *SAMD11* showed significant differences in immune cell infiltration between wild-type and mutation-type. Therefore, we selected them as hub genes for validation of expression level. Using transcriptome data from TCGA, we observed a significantly high expression of these four hub genes in AML patients (**Figures 7A–D**). We also validated the expression levels of hub genes by the validation set GSE114868. As depicted in **Figures 7E–H**, *CDK14* did not show statistical differences between AML and normal tissue, while the expression levels of residual hub genes remained consistent with TCGA. In addition, we validated the expression level of hub genes in AML cell lines. Compared with normal bone marrow mesenchymal stem cells, the relative expression levels of the four hub genes were significantly increased in AML cell lines (**Figures 7I–L**).

## 4 Discussion

Acute myeloid leukemia has a high degree of clinical heterogeneity. Therefore, even patients with the same clinical phenotype may have different outcomes (2). As the relationship between cytogenetics and AML is fully demonstrated, many researchers have devoted themselves to revealing the role of immune factors in the occurrence and development of AML and considering it as a potential prognostic factor (21–23). In this study, we obtained the turquoise module, which is highly correlated with the immune infiltration score, by performing WGCNA on transcriptome data and corresponding clinical information downloaded from the TCGA-LAML database. We also constructed a prognostic model involving eight hub genes through unsupervised clustering, mutation analysis, and LASSO regression analysis. Then, a nomogram was displayed based on the riskscore and age of the prognostic model for survival prediction. Meanwhile, we conducted an enrichment analysis on the turquoise module and two riskscore groups. In addition, we also depicted the immune infiltrating tumor microenvironment landscape of high-risk and low-risk group and hub genes, aiming to decipher the pathogenic role of immune infiltration-related genes in the bone marrow microenvironment of AML. The results of this study indicated that the newly identified immune infiltration-related prognostic model could serve as a potential prognostic biomarker for AML.

The prediction model obtained from high-dimensional data may have the risk of overfitting, and the LASSO penalty effectively addresses this defect (24). So far, several prognostic models have been reported. For example, Guo et al. constructed a prognostic model of six genes closely related to genetic abnormalities (25). However, most previous studies lacked an evaluation of the effectiveness of prognostic models in predicting survival. Compared with previous prognostic models, the prognostic model established in this study exhibited better predictive performance (1-year AUC = 0.787; 3-year AUC = 0.839; 5-year AUC = 0.920). More importantly, we focused on constructing immune infiltration-related prognostic models for the first time, which, to our knowledge, have been rarely reported in AML. In addition, the prognostic model we constructed was not affected by other clinical features and exhibited prognostic independence. Age could also serve as an independent prognostic factor. Next, we combined the risk score and age to construct a nomogram to further reveal the accuracy of survival prediction.

The GO and KEGG enrichment analysis results, indicated that the genes generated from immune factor-related modules and unsupervised clustering were closely related to biological processes involving immune cells and the immune system. According to early reports, *FBXO11* had a tumor-suppressive effect on myeloid malignancies. Its absence led to significant changes in the transcription pathways that affected leukocyte proliferation, differentiation, and apoptosis, thereby inducing MDS to AML transformation (26). Wu et al. found that the frequency of Th17 cells significantly increased in peripheral blood samples of untreated AML patients. When patients achieved complete remission after chemotherapy, the increased frequency of Th17 cells decreased (27). Vegiventi et al. systematically reviewed the dysregulation of the innate immune system and inflammation-related pathways associated with hematopoietic defects in the bone marrow microenvironment, which may affect the progression of AML, as well as the variability in Toll-like receptors (TLRs) expression and NF- κB activation, IL1 receptor-associated kinase (IRAK) dysregulation, the changes of TGF- β and SMAD signaling pathway were both related to the pathogenesis of MDS/AML (28). It was worth noting that the enrichment analysis results between different riskscore groups were highly consistent with the above biological processes, further confirming that our constructed riskscore model could indirectly reflect immune infiltration.

To reveal the complexity of the AML bone marrow microenvironment, we assessed the infiltration levels of immune cells in different groups. In ssGSEA and ImmuCellAI immune infiltration analysis, the infiltration abundance of the DCs, Tem cell (T effector memory), NK cell, and Macrophages group were significantly increased in the high-risk scoring group. Moore et al. confirmed that the bone marrow microenvironment regulated the occurrence, proliferation, and chemotherapy resistance of AML, and the depletion of bone marrow macrophages promoted the growth of AML cells *in vivo (* 29). Macrophages can be roughly divided into two categories: pro-inflammatory and anti-inflammatory. Tumor associated macrophages being commonly anti-inflammatory (30). We know that increased macrophage infiltration is part of the AML immune landscape, probably related to its anti-inflammatory.

Through immune infiltration analysis in the TIMER database, we found that compared with other genes in the prognostic model, *CDK14*, *CRISPLD1*, *MYB*, and *SAMD11* showed significant differences in immune cell infiltration during mutations, indicating the vital role of these genes in the prognostic model. Currently, some studies have reported that these four hub genes in prognostic models play key roles in the tumor microenvironment. Schulz et al. found that the transcription factor *MYB* was essential for macrophage development (31). Zhao et al. confirmed that the *MYB* was the main regulatory factor for hematopoiesis, which can promote proliferation, inhibit cell apoptosis, and block differentiation, tending to the occurrence of leukemia (32). Abnormal expression of *MYB* can lead to changes in immune cell infiltration in the tumor microenvironment. When *MYB* is upregulated, the number of activated cytotoxic CD8+ T cells increase, helping to regulate tumor growth (33). Stromal cells in the tumor microenvironment are mainly composed of Cancer-associated fibroblasts (CAFs). As an important member of CAFs-related genes, *CDK14* can predict the effect of anti-PD-1 treatment in melanoma patients (34). Park et al. identified genetic changes in *CRISPLD1* in renal cell carcinoma (RCC) through whole exome sequencing (WES) (35). As a member of the cysteine-rich secretory proteins, antigen 5, and pathogenesis-related 1 proteins (CAP) family, *CRISPLD1* was found to be closely related to tumors and immune defense (36). Titus et al. identified the abnormal methylation site of *SAMD11* in breast cancer through the TCGA database (37). Unfortunately, there is no literature documenting that *SAMD11* is related to the tumor immune microenvironment. In short, these results indicate that hub genes play an important role in the tumor microenvironment (or bone marrow microenvironment). Although the functions of these hub genes in AML have not been fully elucidated, we still confirmed their role in prognostic models.

The RT-qPCR results of AML cells showed significantly high *CDK14*, *CRISPLD1*, *MYB*, and *SAMD11* expression in HL-60, THP-1, and MOLM-13 cells, consistent with our bioinformatics results. Based on previous research and the results of this study, we speculated that changes in the pathological process of AML may create a specific immune microenvironment that can recruit various immune cells, including T cell, DC, and macrophage populations. The recruited cells interacted with AML cells through various immune-related signaling pathways and cytokines and led to abnormal expression of immune infiltration-related genes involved in the LASSO model. They, in turn, promoted the progression of AML.

The advantage of this study lies in the application of multiple bioinformatics methods to systematically analyze the transcriptome data of TCGA-LAML, providing essential insights into the immune infiltration-related genes (IIRGs) driving immune cell infiltration into the bone marrow microenvironment of AML. Despite the excellent risk stratification and predictive performance of the prognostic model constructed of 8 hub genes, our study still had certain limitations. On the one hand, the prognostic model constructed with eight hub genes cannot fully represent the transcriptional expression profile landscape of the entire AML genome, and the prognostic model we constructed lacked prognostic efficacy evaluation of a large number of clinical samples. On the other hand, we only validated the relative expression levels of hub genes in AML cell lines, needing more in-depth mechanism research *in vivo* and *in vitro*. Although we know that there are these limitations, this type of modelling is powerful to identify gene targets for further studies. In the future, we will explore the mechanisms by which immune infiltration-related genes promote AML progression through *in vivo* and *in vitro* experiments. At the same time, we will also apply prognostic models to clinical trials to further evaluate the robustness of their prognostic efficacy and achieve clinical translation.

## 5 Conclusion

In summary, we applied WGCNA, unsupervised clustering, and mutation analysis to identify 17 candidate genes with high mutation frequencies and clinical significance. Subsequently, a prognostic model with reasonable risk stratification performance was established using the LASSO algorithm. The prognostic model we constructed exhibits excellent predictive performance compared to traditional prognostic evaluation methods. We also identified a nomogram that combines riskscore and age for further survival prediction. In addition, a series of immune infiltration analyses revealed that abnormal infiltration of immune cells into the bone marrow microenvironment driven by hub genes and high-risk score groups may be a prominent immune landscape in the occurrence and development of AML. To sum up, the prognostic model we have constructed involving immune infiltration-related genes is expected to become a potential prognostic marker for AML.

## Data availability statement

The raw data supporting the conclusions of this article will be made available by the authors, without undue reservation.

## Ethics statement

Ethical approval was not required for the studies on humans in accordance with the local legislation and institutional requirements because only commercially available established cell lines were used.

## Author contributions

JJ: Conceptualization, Writing – original draft, Writing – review & editing. SY: Formal analysis, Software, Validation, Writing – review & editing.

## Supplementary material

Supplementary Figure 1 Flow chart of the present study.

Supplementary Figure 2 The scale-free fit index analysis and functional enrichment analysis. **(A)**. The scale-free fit index analysis and the mean connectivity for various soft-thresholding powers (β). **(B–E)**. Bubble chart of GO and KEGG functional enrichment analysis in turquoise module.

Supplementary Figure 3 Unsupervised clustering analysis. **(A)**. Consensus clustering cumulative distribution function (CDF) for k=2 to 7. **(B)**. Relative change in area under CDF curve for k=2 to 7. **(C)**. Uniform Manifold Approximation and Projection (UMAP) analysis of two groups. X-axis represents the UMAP1 and Y-axis represents UMAP2. **(D)**. t-distributed Stochastic Neighbor Embedding (t-SNE) analysis of two groups. X-axis represents the tSNE1 and Y-axis represents tSNE2.

Supplementary Figure 4 The landscape of mutation analysis. **(A)**. The top 10 mutated genes. **(B)**. Classification and frequency of mutation types. **(C)**. Frequency of variant types. **(D)**. Frequency of SNV class. **(E, F)**. Tumor mutation burden in specific samples.

Supplementary Figure 5 Kaplan–Meier survival analysis for 8 genes based on LASSO prognosis model.

Supplementary Figure 6 Analysis of risk scores with clinical characteristics. **(A–P)**. Relationship between age, gender, WBC counts, chromosome abnormality and gene mutation with the risk score.

Supplementary Figure 7 Validation of the relationship between the mutation of genes and the infiltration level of immune cells in TIMER database.

Supplementary Figure 8 Immune infiltration analysis of high-risk and low-risk groups. **(A)**. Heatmap of immune infiltration analysis in the “ssGSEA” algorithm. **(B)**. Heatmap of immune infiltration analysis in the “ImmuCellAI” algorithm. **(C)**. Violin plot of immune infiltration analysis in the “ssGSEA” algorithm. **(D)**. Violin plot of immune infiltration analysis in the “ImmuCellAI” algorithm.
