# Supplementary figures and images for: Immune infiltration-related genes regulate the progression of AML by invading the bone marrow microenvironment
Source: Front Immunol. 2024 Jul 12;15:1409945. doi: 10.3389/fimmu.2024.1409945 (PMC11272452; doi:10.3389/fimmu.2024.1409945)

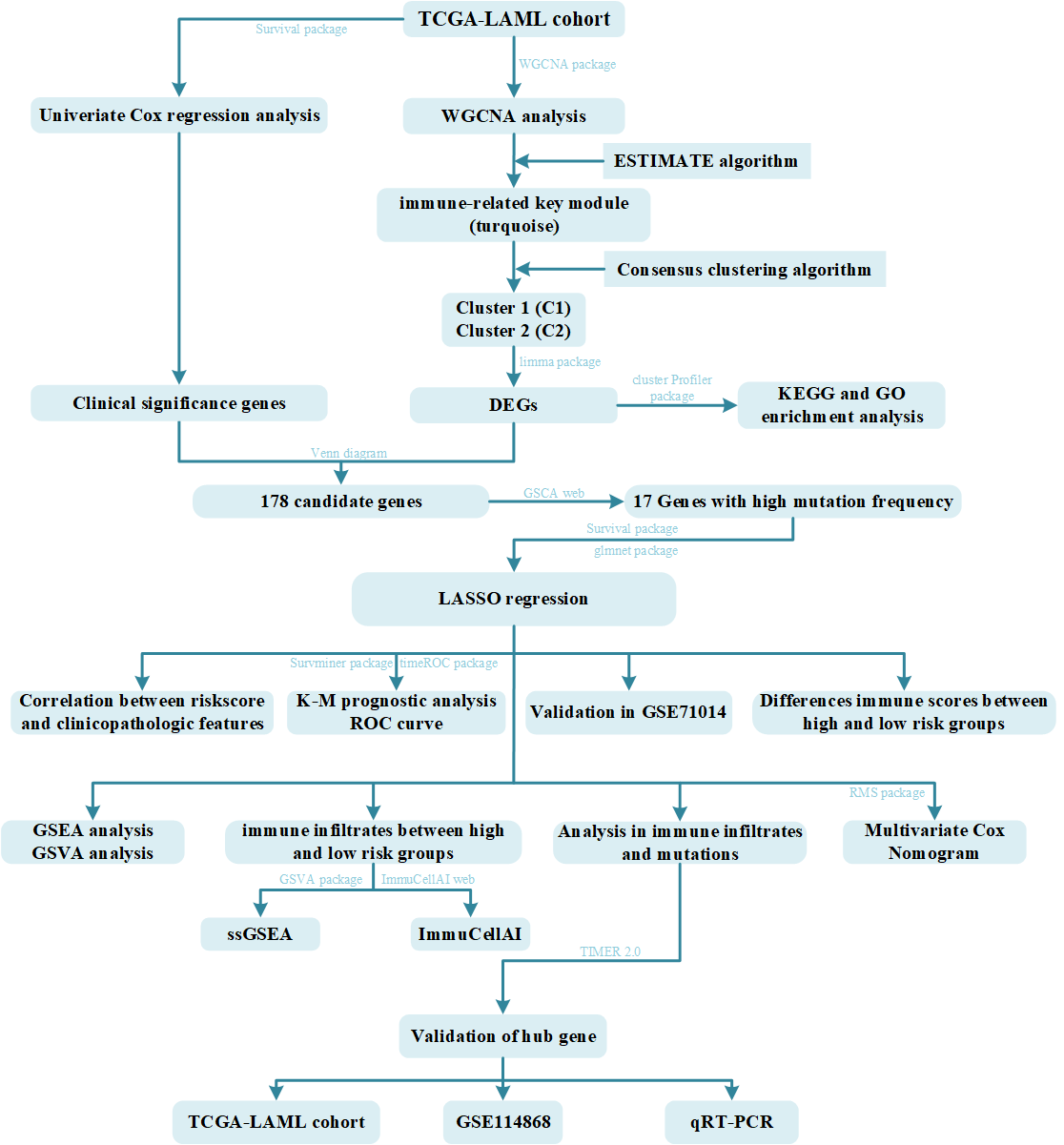

Supplement: Supplementary Figure 1 — Flow chart of the present study. [file Image_1.tif]

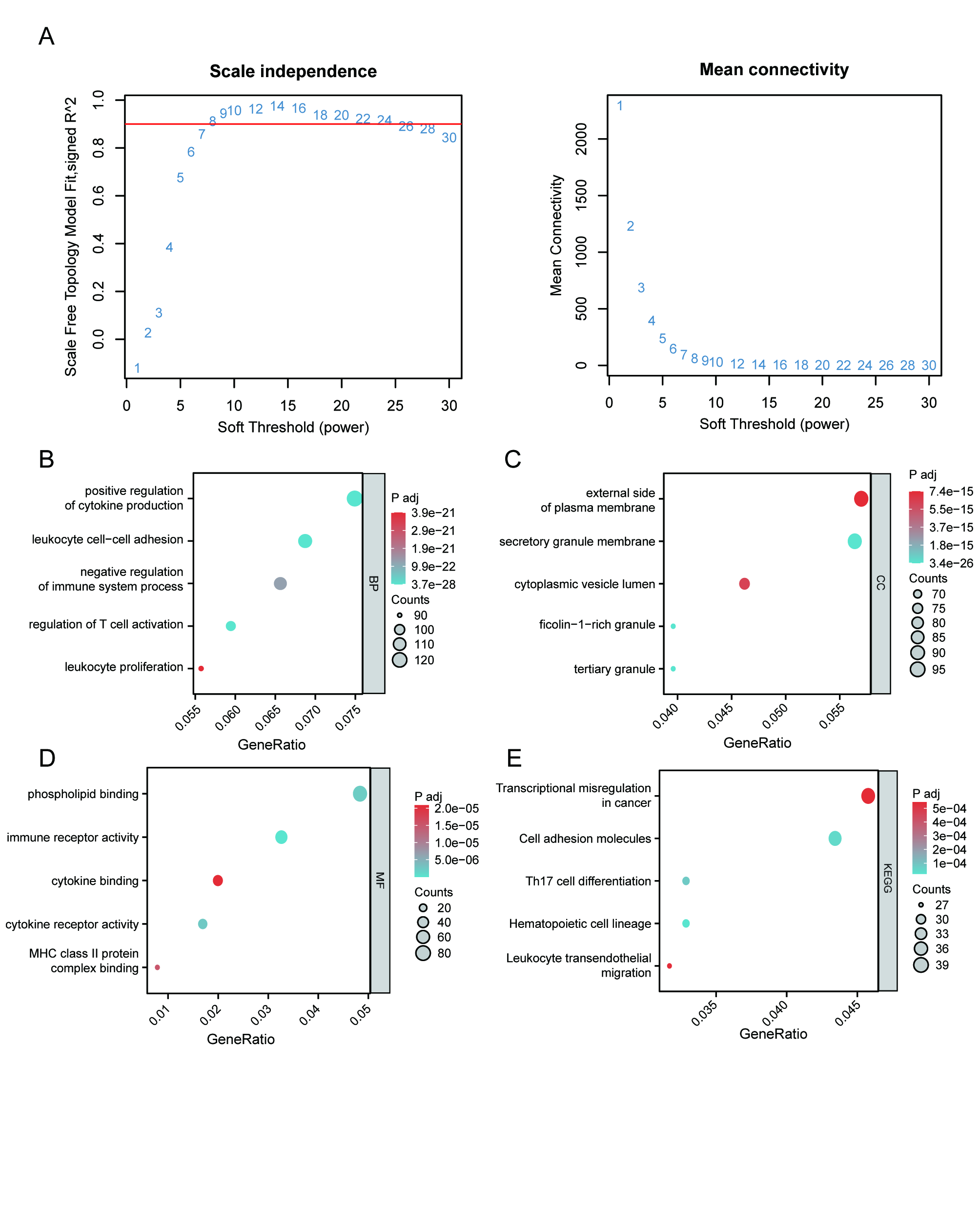

Supplement: Supplementary Figure 2 — The scale-free fit index analysis and functional enrichment analysis. (A). The scale-free fit index analysis and the mean connectivity for various soft-thresholding powers (β). (B–E). Bubble chart of GO and KEGG functional enrichment analysis in turquoise module. [file Image_2.tif]

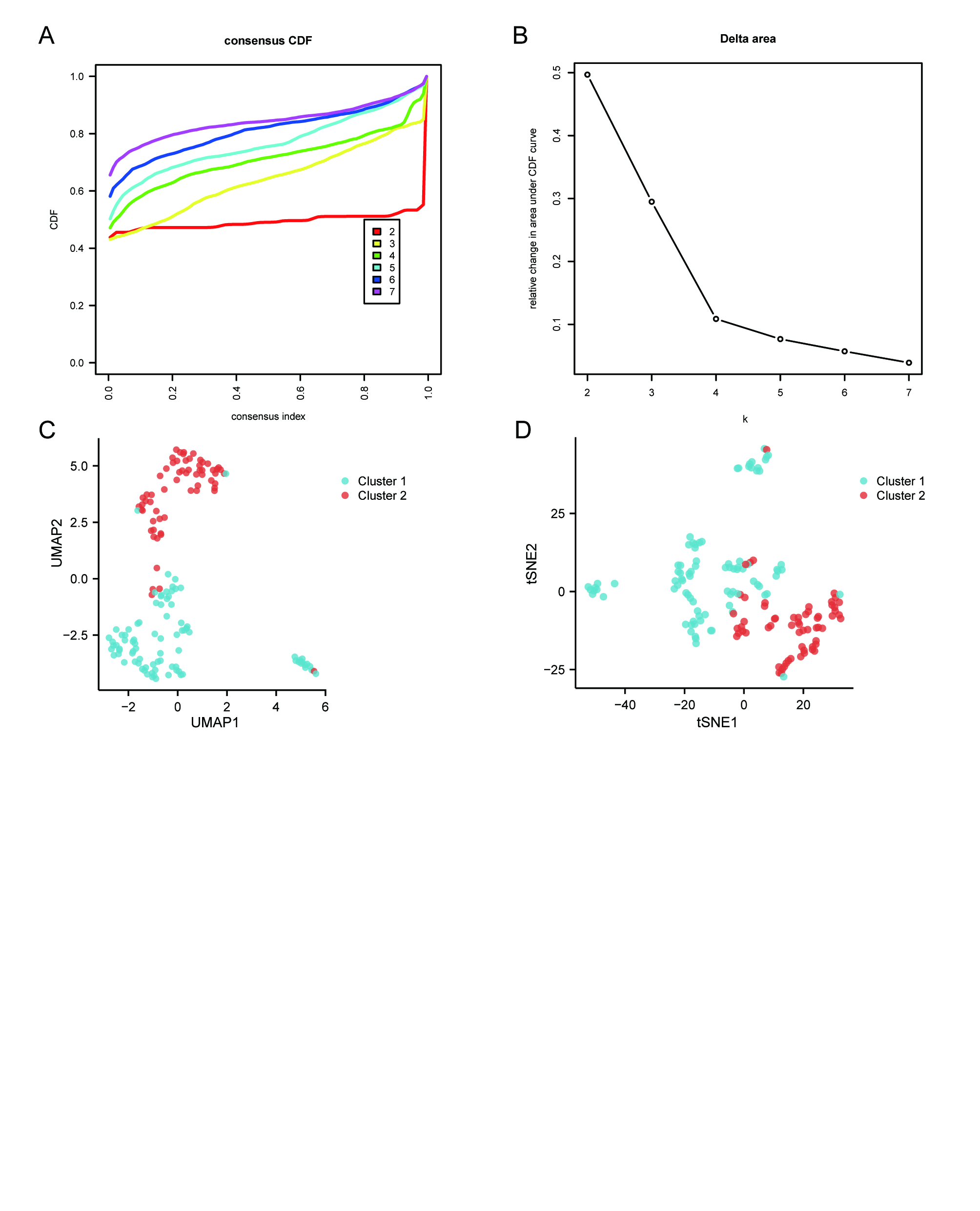

Supplement: Supplementary Figure 3 — Unsupervised clustering analysis. (A). Consensus clustering cumulative distribution function (CDF) for k=2 to 7. (B). Relative change in area under CDF curve for k=2 to 7. (C). Uniform Manifold Approximation and Projection (UMAP) analysis of two groups. X-axis represents the UMAP1 and Y-axis represents UMAP2. (D). t-distributed Stochastic Neighbor Embedding (t-SNE) analysis of two groups. X-axis represents the tSNE1 and Y-axis represents tSNE2. [file Image_3.tif]

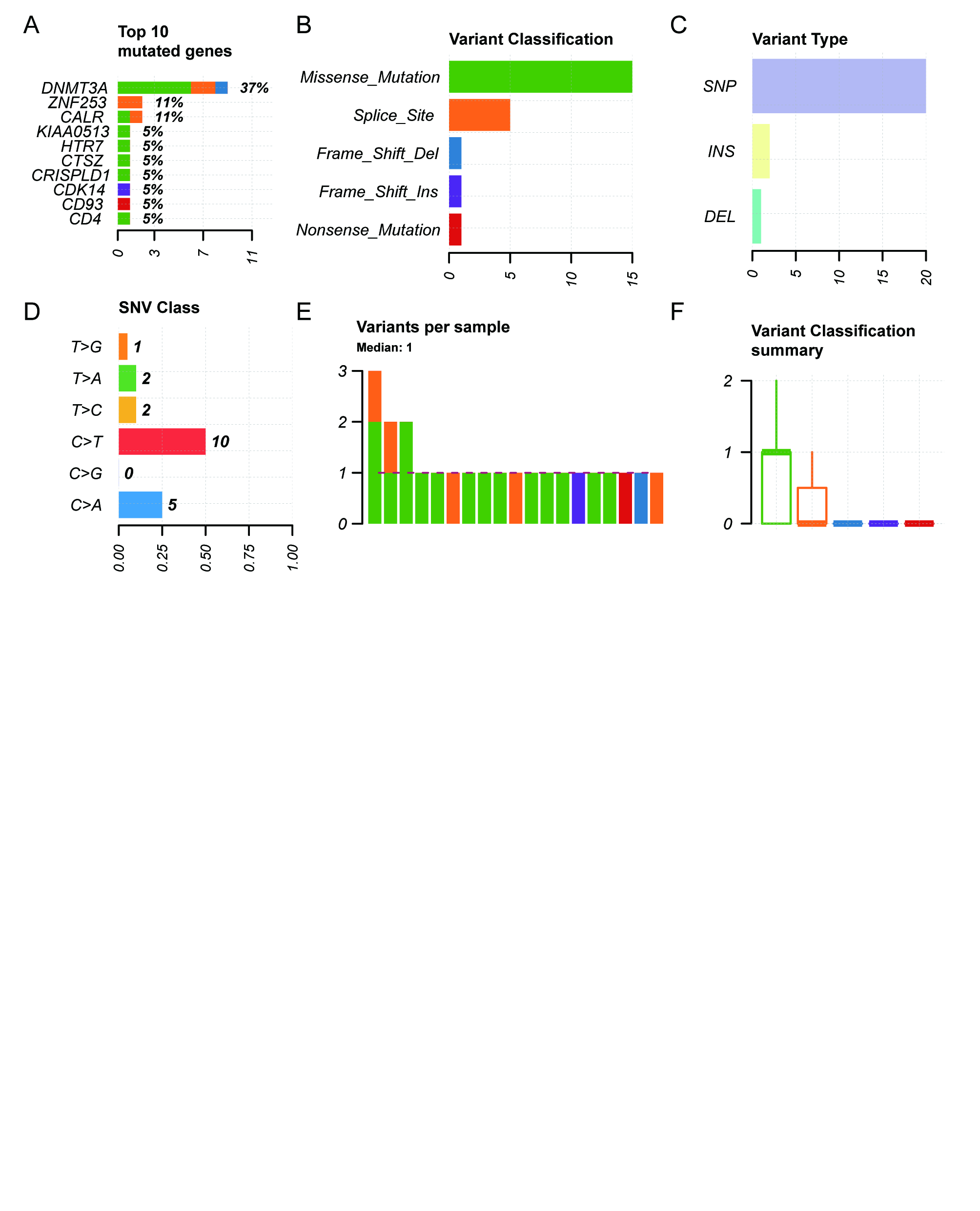

Supplement: Supplementary Figure 4 — The landscape of mutation analysis. (A). The top 10 mutated genes. (B). Classification and frequency of mutation types. (C). Frequency of variant types. (D). Frequency of SNV class. (E, F). Tumor mutation burden in specific samples. [file Image_4.tif]

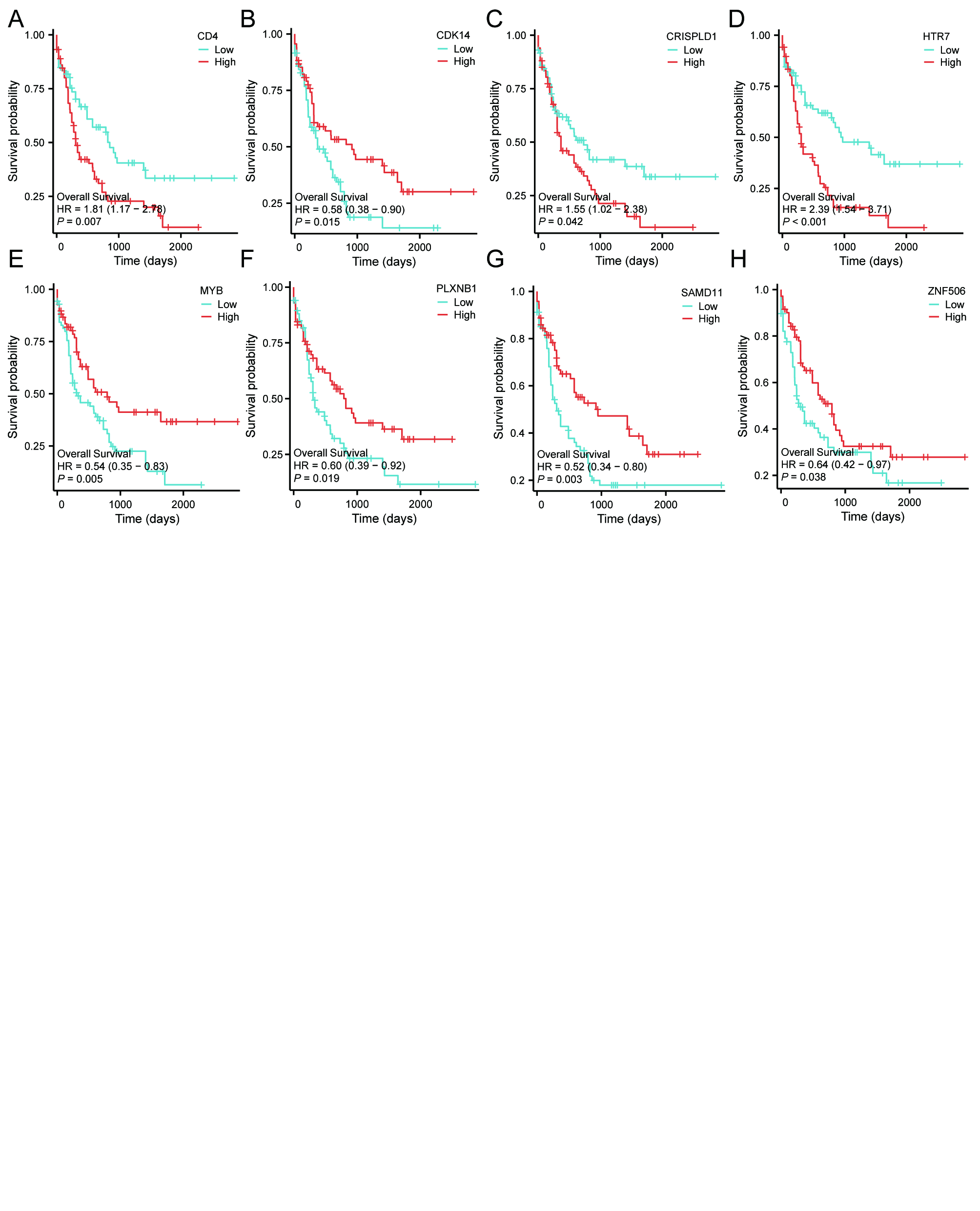

Supplement: Supplementary Figure 5 — Kaplan–Meier survival analysis for 8 genes based on LASSO prognosis model. [file Image_5.tif]

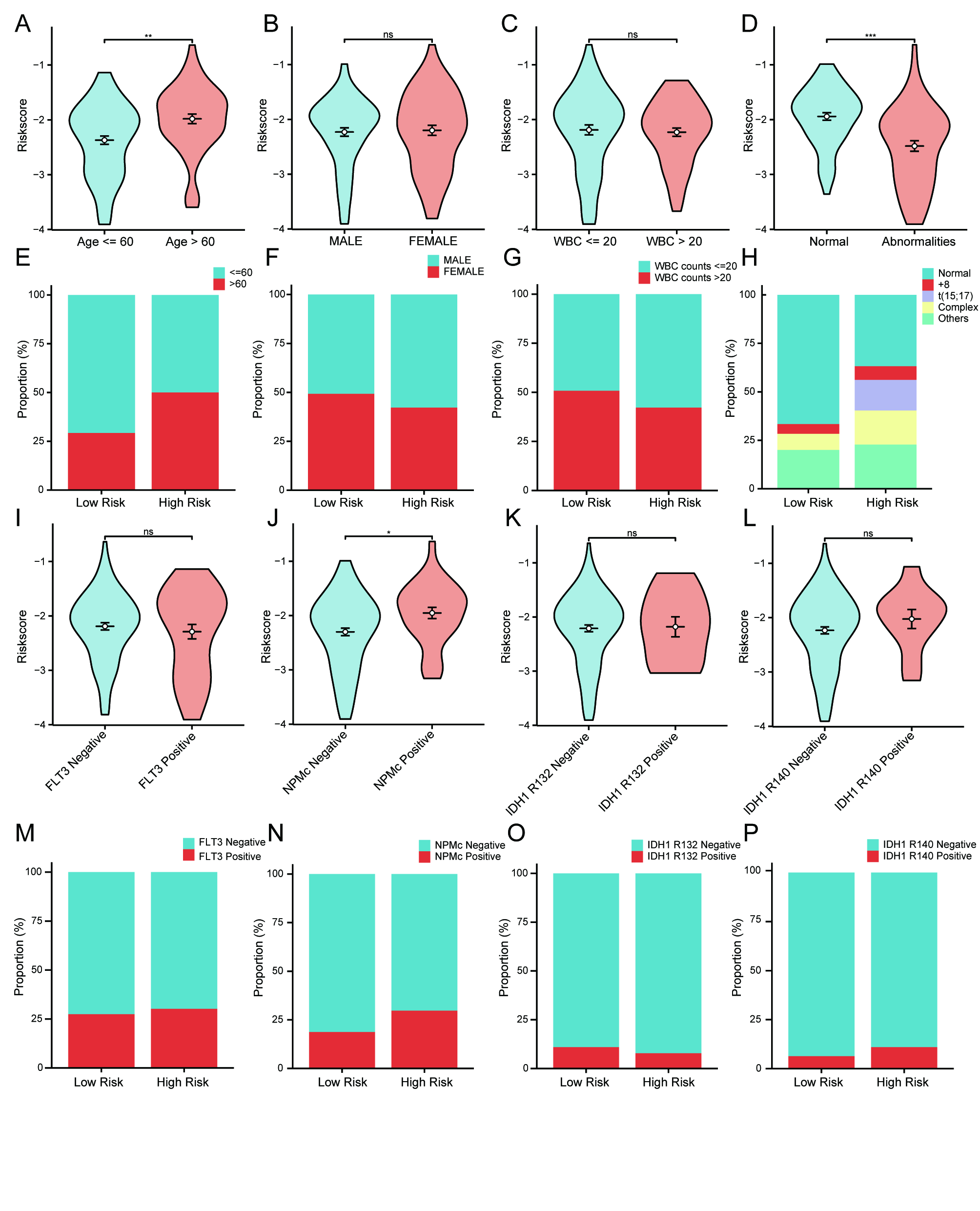

Supplement: Supplementary Figure 6 — Analysis of risk scores with clinical characteristics. (A–P). Relationship between age, gender, WBC counts, chromosome abnormality and gene mutation with the risk score. [file Image_6.tif]

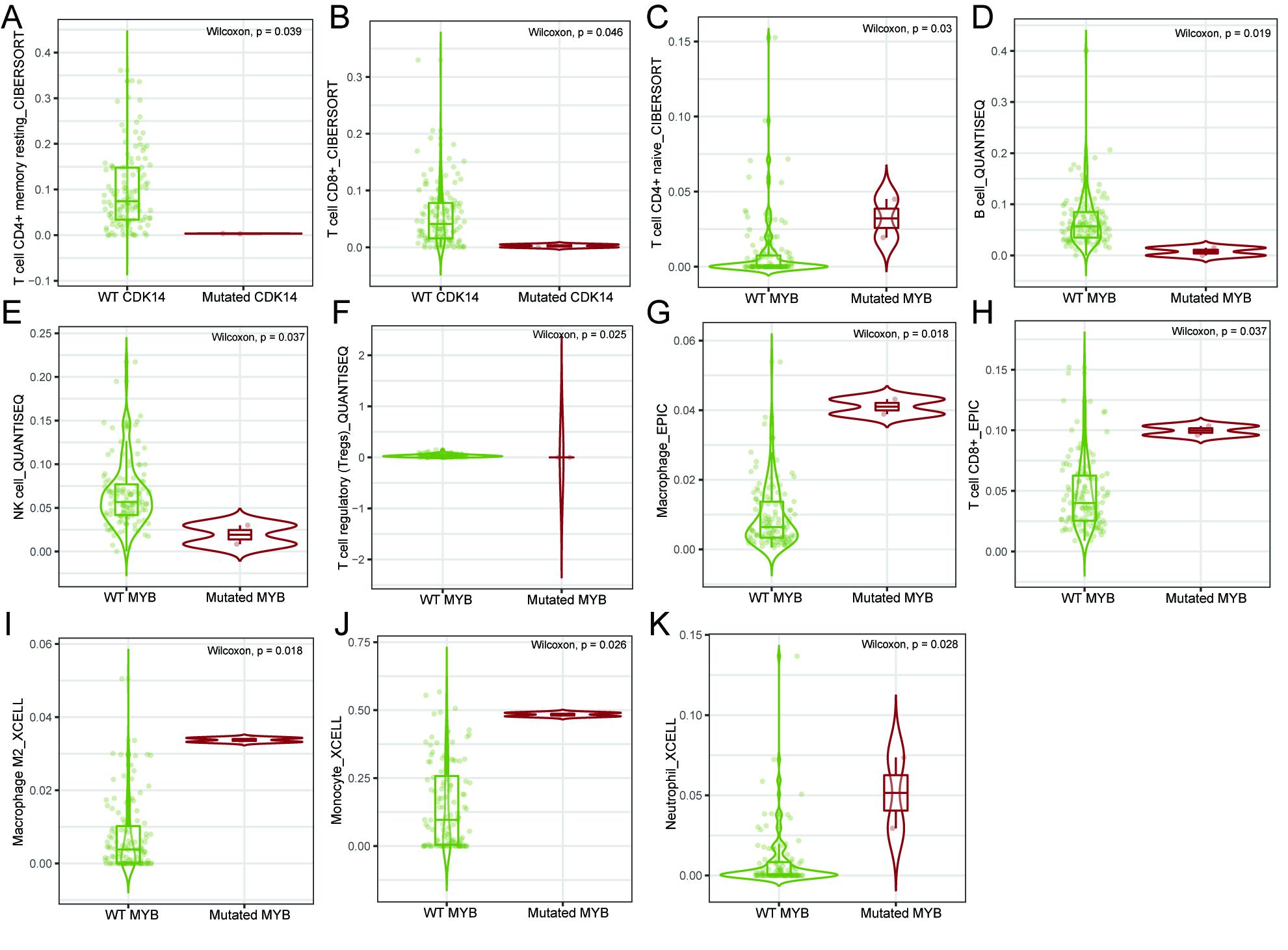

Supplement: Supplementary Figure 7 — Validation of the relationship between the mutation of genes and the infiltration level of immune cells in TIMER database. [file Image_7.tif]

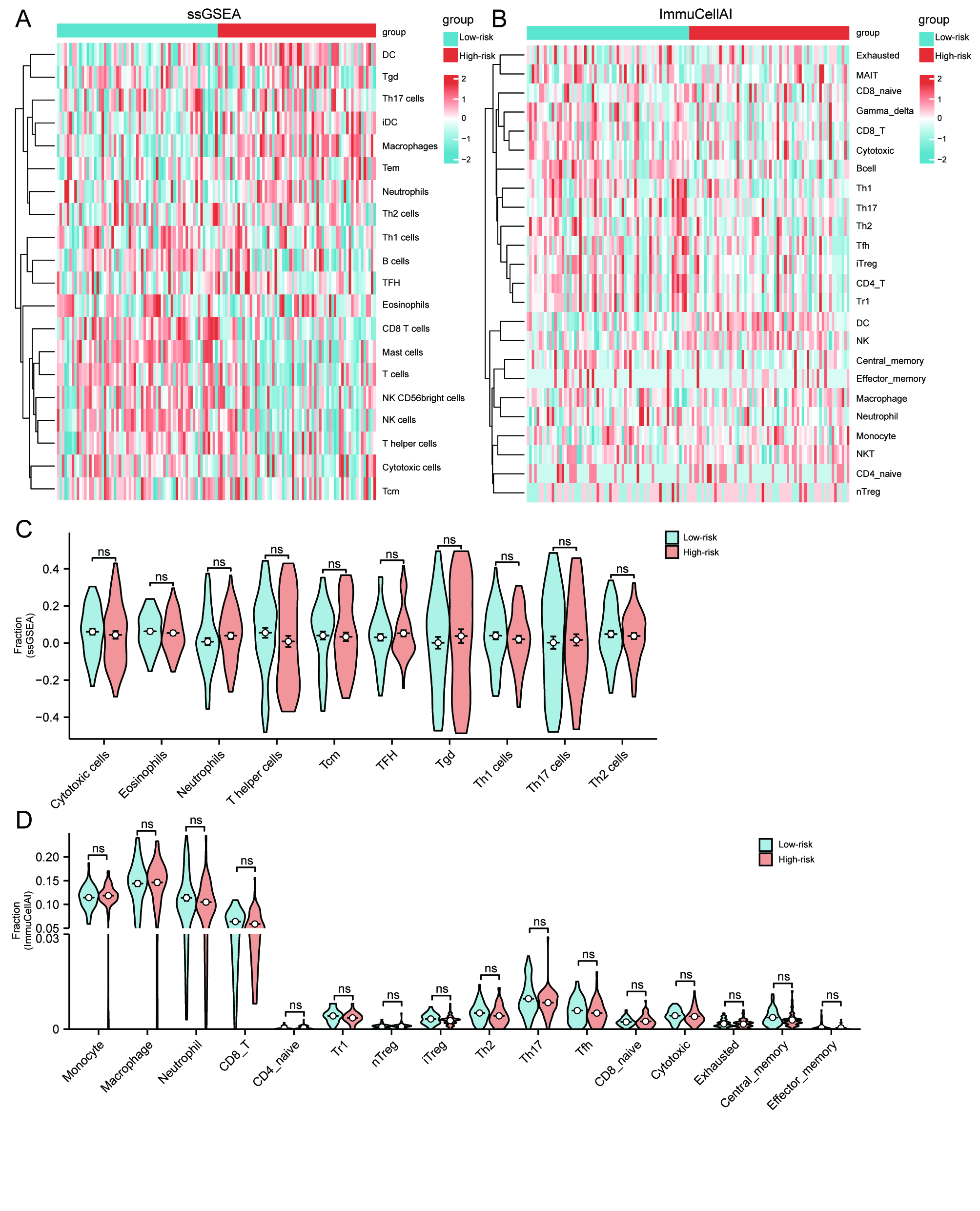

Supplement: Supplementary Figure 8 — Immune infiltration analysis of high-risk and low-risk groups. (A). Heatmap of immune infiltration analysis in the “ssGSEA” algorithm. (B). Heatmap of immune infiltration analysis in the “ImmuCellAI” algorithm. (C). Violin plot of immune infiltration analysis in the “ssGSEA” algorithm. (D). Violin plot of immune infiltration analysis in the “ImmuCellAI” algorithm. [file Image_8.tif]
