# Supplementary material for: Immune infiltration-related genes regulate the progression of AML by invading the bone marrow microenvironment
Source: Front Immunol. 2024 Jul 12;15:1409945. doi: 10.3389/fimmu.2024.1409945 (PMC11272452; doi:10.3389/fimmu.2024.1409945)
Supplement: Supplementary file 9 [file Table_1.docx]

Table S1. The detailed information of GEO database

| **Data source** | **series** | **Platform** | **Author** | **Year** | **Samples (T&N)** | **Tissue** |
| --- | --- | --- | --- | --- | --- | --- |
| mRNA | GSE71014 | GPL10558 | Chou W | 2015 | 104/0 | Bone marrow |
| mRNA | GSE114868 | GPL17586 | Huang H | 2018 | 194/20 | Bone marrow |
