# Supplementary material for: Immune infiltration-related genes regulate the progression of AML by invading the bone marrow microenvironment
Source: Front Immunol. 2024 Jul 12;15:1409945. doi: 10.3389/fimmu.2024.1409945 (PMC11272452; doi:10.3389/fimmu.2024.1409945)
Supplement: Supplementary file 10 [file Table_2.docx]

Table S2. Primers of mRNAs in present study

| **mRNA** | **Primer (5’- 3’)** |
| --- | --- |
| MYB |  |
|  | 5’- TGAGGACTTTGAGATGTGTG -3’ |
|  | 5’- GTCATCTGTTCCATTCTGTTC -3’. |
| CDK14 |  |
|  | 5’- TCTTTACCACATTTTAAGCC -3’ |
|  | 5’- GGGAACATTGTAGGAGCTTGG -3’ |
| CRISPLD1 |  |
|  | 5’- GAAACAAATGAAATAGAACG -3’ |
|  | 5’- TGCTTTCTTCCTTGTCTAGTG -3’ |
| SAMD11 |  |
|  | 5’- TTCCCTTATGCCGTCAGCCCC -3’ |
|  | 5’- GTCGATCCCCTGCTCCCTGAA -3’ |
| GAPDH |  |
|  | 5’- CATGTTCGTCATGGGTGTGAA -3’ |
|  | 5’- GGCATGGACTGTGGTCATGAG -3’ |
