# Supplementary material for: Immune infiltration-related genes regulate the progression of AML by invading the bone marrow microenvironment
Source: Front Immunol. 2024 Jul 12;15:1409945. doi: 10.3389/fimmu.2024.1409945 (PMC11272452; doi:10.3389/fimmu.2024.1409945)
Supplement: Supplementary file 11 [file Table_3.docx]

| Table S3. GO and KEGG enrichment analysis of turquoise module | | | | | |
| --- | --- | --- | --- | --- | --- |
| **ID** | **Description** | **Count** | **p-value** | **FDR** | **mRNAs** |
| **BP terms** |  |  |  |  |  |
| GO:0007159 | leukocyte cell-cell adhesion | 111 | 3.71E-28 | 2.68E-28 | ADA, ADAM8, AIF1, ALOX5, BCL6, BTN2A2, CASP3, CCDC88B, CCR2, CD1D, CD276, CD300A, CD4, CD55, CD70, CD74, CD83, CD86, CEBPB, CERCAM, CHST2, CORO1A, CR1, CX3CR1, EXT1, F11R, FCGR2B, FGL2, FYN, HAVCR2, HFE, HHLA2, HLA-DMB, HLA-DPA1, HLA-DPB1, HLA-DQA1, HLA-DQB1, HLA-DQB2, HLA-DRA, HLA-DRB1, HLA-DRB5, HLA-E, HLX, ICAM1, IGFBP2, IL10, IL12RB1, IL15, IL4R, IL6ST, ITGA4, ITGAL, ITGB2, ITGB7, JAK2, KLF4, LAPTM5, LEP, LGALS1, LGALS3, LILRB1, LILRB2, LILRB4, LOXL3, LYN, MDK, MYB, NDFIP1, NFAT5, NFKBIZ, NLRP3, NOD2, OLR1, PAG1, PECAM1, PELI1, PRKCQ, PRNP, PTAFR, PTPN6, PTPRC, PYCARD, RARA, RHOH, RUNX1, S100A8, S100A9, SASH3, SDC4, SELPLG, SIRPA, SIRPB1, SLAMF1, SLC7A1, SOX12, SOX4, SPN, SRC, ST3GAL4, TESPA1, TGFBR2, TNFSF13B, TNFSF14, TNFSF9, VNN1, VSIG4, XBP1, YES1, ZBTB16, ZBTB7B, ZMIZ1 |
| GO:0001819 | positive regulation of cytokine production | 121 | 3.22E-25 | 2.33E-25 | ADAM8, ADRA2A, AIF1, AIM2, APOA2, BCL3, BMPR1A, C3, C3AR1, C5AR1, CADM1, CARD9, CASP1, CCDC88B, CCR2, CD14, CD276, CD36, CD4, CD55, CD74, CD83, CD86, CEBPB, CLEC4E, CLEC7A, CSF1R, CYBA, CYBB, EPHB2, F2R, FCER1G, FCN1, FFAR2, FGR, FLOT1, FZD5, GPSM3, HAVCR2, HHLA2, HK1, HLA-DPA1, HLA-DPB1, HLA-E, HMOX1, HPSE, HSPA1A, IFNGR1, IL10, IL12RB1, IL15, IL17RA, IL4R, IL6R, IL6ST, IRAK3, IRF5, IRF7, IRF8, JAK2, LAPTM5, LEP, LILRA2, LILRA5, LILRB1, LILRB2, LRRK2, LY96, MDK, MEFV, MMP8, MNDA, MYD88, NFAM1, NLRC4, NLRP1, NLRP12, NLRP3, NOD2, OAS1, OAS2, OSM, P2RX7, PELI1, POU2F2, PRKCQ, PTAFR, PTGS2, PTPRC, PTPRJ, PYCARD, RAB7B, RARA, RUNX1, SASH3, SCIMP, SERPINE1, SLAMF1, SLC11A1, SLC7A5, SPN, SPTBN1, SRC, SULF2, TGFB1, THBS1, TLR1, TLR2, TLR4, TLR5, TLR6, TLR7, TLR8, TNFRSF8, TRIM6, TXK, TYROBP, XBP1, ZBTB20, ZBTB7B, ZFPM1 |
| GO:0022409 | positive regulation of cell-cell adhesion | 90 | 5.39E-25 | 3.89E-25 | ADA, ADAM8, AIF1, ALOX5, BCL6, BTN2A2, CCDC88B, CCR2, CD1D, CD276, CD4, CD55, CD70, CD74, CD83, CD86, CHST2, CORO1A, CR1, EMILIN2, F11R, FLOT1, FSTL3, FYN, GCNT2, HAVCR2, HHLA2, HLA-DMB, HLA-DPA1, HLA-DPB1, HLA-DQA1, HLA-DQB1, HLA-DQB2, HLA-DRA, HLA-DRB1, HLA-DRB5, HLA-E, HLX, IGFBP2, IL10, IL12RB1, IL15, IL4R, IL6ST, ITGA4, ITGB2, JAK2, KIF26B, KIFAP3, LEP, LGALS1, LILRB1, LILRB2, LILRB4, LYN, MDK, MYB, NFAT5, NFKBIZ, NLRP3, NOD2, PLAUR, PRKCQ, PTAFR, PTPN6, PTPRC, PYCARD, RARA, RHOH, RUNX1, SASH3, SIRPA, SIRPB1, SLAMF1, SLC7A1, SOX12, SOX4, SRC, ST3GAL4, TESPA1, TGFBR2, TNFSF13B, TNFSF14, TNFSF9, VNN1, XBP1, YES1, ZBTB16, ZBTB7B, ZMIZ1 |
| GO:1903037 | regulation of leukocyte cell-cell adhesion | 99 | 5.39E-25 | 3.89E-25 | ADA, ADAM8, AIF1, ALOX5, BCL6, BTN2A2, CASP3, CCDC88B, CCR2, CD1D, CD276, CD300A, CD4, CD55, CD70, CD74, CD83, CD86, CEBPB, CHST2, CORO1A, CR1, FCGR2B, FGL2, FYN, HAVCR2, HFE, HHLA2, HLA-DMB, HLA-DPA1, HLA-DPB1, HLA-DQA1, HLA-DQB1, HLA-DQB2, HLA-DRA, HLA-DRB1, HLA-DRB5, HLA-E, HLX, IGFBP2, IL10, IL12RB1, IL15, IL4R, IL6ST, ITGA4, ITGB2, JAK2, KLF4, LAPTM5, LEP, LGALS1, LGALS3, LILRB1, LILRB2, LILRB4, LOXL3, LYN, MDK, MYB, NDFIP1, NFAT5, NFKBIZ, NLRP3, NOD2, PAG1, PELI1, PRKCQ, PRNP, PTAFR, PTPN6, PTPRC, PYCARD, RARA, RHOH, RUNX1, SASH3, SDC4, SIRPA, SIRPB1, SLAMF1, SLC7A1, SOX12, SOX4, SPN, SRC, ST3GAL4, TESPA1, TGFBR2, TNFSF13B, TNFSF14, TNFSF9, VNN1, VSIG4, XBP1, YES1, ZBTB16, ZBTB7B, ZMIZ1 |
| GO:0022407 | regulation of cell-cell adhesion | 117 | 5.39E-25 | 3.89E-25 | ADA, ADAM8, AIF1, ALOX5, BCL6, BTN2A2, CASP3, CCDC88B, CCR2, CD1D, CD276, CD300A, CD4, CD55, CD70, CD74, CD83, CD86, CEBPB, CHST2, CORO1A, CR1, EMILIN2, EPCAM, EPHB3, F11R, FCGR2B, FGL2, FLOT1, FSTL3, FYN, GCNT2, HAVCR2, HFE, HHLA2, HLA-DMB, HLA-DPA1, HLA-DPB1, HLA-DQA1, HLA-DQB1, HLA-DQB2, HLA-DRA, HLA-DRB1, HLA-DRB5, HLA-E, HLX, IGFBP2, IL10, IL12RB1, IL15, IL1RN, IL4R, IL6ST, ITGA4, ITGB2, JAK2, KIF26B, KIFAP3, KLF4, LAPTM5, LEP, LGALS1, LGALS3, LILRB1, LILRB2, LILRB4, LOXL3, LYN, MAPK7, MDGA1, MDK, MYB, NDFIP1, NFAT5, NFKBIZ, NLRP3, NOD2, PAG1, PELI1, PLAUR, PRKCA, PRKCD, PRKCQ, PRNP, PTAFR, PTPN6, PTPRC, PYCARD, RARA, RHOH, RUNX1, SASH3, SDC4, SIRPA, SIRPB1, SLAMF1, SLC7A1, SOX12, SOX4, SPI1, SPN, SRC, ST3GAL4, TESPA1, TGFB1, TGFBR2, TNFSF13B, TNFSF14, TNFSF9, VNN1, VSIG4, XBP1, YES1, ZBTB16, ZBTB7B, ZMIZ1, ZNF703 |
| GO:1903039 | positive regulation of leukocyte cell-cell adhesion | 81 | 1.11E-24 | 8.04E-25 | ADA, ADAM8, AIF1, ALOX5, BCL6, BTN2A2, CCDC88B, CCR2, CD1D, CD276, CD4, CD55, CD70, CD74, CD83, CD86, CHST2, CORO1A, CR1, FYN, HAVCR2, HHLA2, HLA-DMB, HLA-DPA1, HLA-DPB1, HLA-DQA1, HLA-DQB1, HLA-DQB2, HLA-DRA, HLA-DRB1, HLA-DRB5, HLA-E, HLX, IGFBP2, IL12RB1, IL15, IL4R, IL6ST, ITGA4, ITGB2, JAK2, LEP, LGALS1, LILRB1, LILRB2, LILRB4, LYN, MDK, MYB, NFAT5, NFKBIZ, NLRP3, NOD2, PRKCQ, PTAFR, PTPN6, PTPRC, PYCARD, RARA, RHOH, RUNX1, SASH3, SIRPA, SIRPB1, SLAMF1, SLC7A1, SOX12, SOX4, SRC, ST3GAL4, TESPA1, TGFBR2, TNFSF13B, TNFSF14, TNFSF9, VNN1, XBP1, YES1, ZBTB16, ZBTB7B, ZMIZ1 |
| GO:0050863 | regulation of T cell activation | 96 | 1.9E-23 | 1.37E-23 | ADA, ADAM8, AIF1, BCL6, BTN2A2, CASP3, CCDC88B, CCR2, CD1D, CD276, CD300A, CD4, CD55, CD70, CD74, CD83, CD86, CEBPB, CORO1A, CR1, FCGR2B, FGL2, FYN, HAVCR2, HFE, HHLA2, HLA-DMB, HLA-DPA1, HLA-DPB1, HLA-DQA1, HLA-DQB1, HLA-DQB2, HLA-DRA, HLA-DRB1, HLA-DRB5, HLA-E, HLX, IGFBP2, IL10, IL12RB1, IL15, IL4R, IL6ST, JAK2, LAPTM5, LEP, LGALS1, LGALS3, LILRB1, LILRB2, LILRB4, LOXL3, LYN, MDK, MYB, NDFIP1, NFKBIZ, NLRP3, NOD2, PAG1, PELI1, PRKCQ, PRNP, PTPN6, PTPRC, PYCARD, RARA, RHOH, RUNX1, SASH3, SDC4, SIRPA, SIRPB1, SLAMF1, SLC46A2, SLC7A1, SOX12, SOX4, SPN, SRC, TESPA1, TGFBR2, TNFRSF1B, TNFSF13B, TNFSF14, TNFSF8, TNFSF9, TOX, VNN1, VSIG4, XBP1, YES1, ZBTB16, ZBTB7B, ZEB1, ZMIZ1 |
| GO:0050870 | positive regulation of T cell activation | 74 | 1.25E-22 | 9.02E-23 | ADA, ADAM8, AIF1, BCL6, BTN2A2, CCDC88B, CCR2, CD1D, CD276, CD4, CD55, CD70, CD74, CD83, CD86, CORO1A, CR1, FYN, HAVCR2, HHLA2, HLA-DMB, HLA-DPA1, HLA-DPB1, HLA-DQA1, HLA-DQB1, HLA-DQB2, HLA-DRA, HLA-DRB1, HLA-DRB5, HLA-E, HLX, IGFBP2, IL12RB1, IL15, IL4R, IL6ST, JAK2, LEP, LGALS1, LILRB1, LILRB2, LILRB4, LYN, MDK, MYB, NFKBIZ, NLRP3, NOD2, PRKCQ, PTPN6, PTPRC, PYCARD, RARA, RHOH, RUNX1, SASH3, SIRPA, SIRPB1, SLAMF1, SLC7A1, SOX12, SOX4, SRC, TESPA1, TGFBR2, TNFSF13B, TNFSF14, TNFSF9, VNN1, XBP1, YES1, ZBTB16, ZBTB7B, ZMIZ1 |
| GO:0002683 | negative regulation of immune system process | 106 | 1.25E-21 | 9.04E-22 | ADA, AHR, APOA2, ARRB2, BCL6, BCR, BPI, BST2, BTN2A2, C5AR2, CASP3, CCR2, CD200R1, CD300A, CD300LF, CD55, CD59, CD74, CD86, CD96, CDK6, CEBPB, CLEC12B, CR1, CX3CR1, DUSP1, FCGR2B, FGL2, FGR, FSTL3, FYN, GATA2, GPR137B, GPX1, GRN, HAVCR2, HCK, HFE, HLA-B, HLA-DRB1, HLA-E, HLX, HMOX1, ID2, IL10, IL31RA, IL4R, IRAK3, LAPTM5, LDLR, LGALS3, LILRB1, LILRB2, LILRB3, LILRB4, LOXL3, LPXN, LST1, LYN, MAFB, MDK, MILR1, MNDA, MYC, NDFIP1, NLRC3, NOD2, OAS1, PADI2, PAG1, PARP14, PELI1, PLCL2, PLK2, PPARG, PRNP, PTPN6, PTPRC, PTPRJ, RARA, RHBDF2, RUNX1, SAMHD1, SAMSN1, SDC4, SERPINB9, SLA2, SLAMF1, SPI1, SPN, SRC, SYT11, TGFB1, THBS1, TLR4, TMEM176A, TMEM176B, TNFAIP3, TNFAIP6, TRIB1, TYROBP, VSIG4, YES1, ZBTB46, ZBTB7B, ZFPM1 |
| GO:0046651 | lymphocyte proliferation | 85 | 1.93E-21 | 1.39E-21 | ADA, AHR, AIF1, ARMC5, AZI2, BCL2, BCL6, BST1, BTN2A2, CASP3, CCDC88B, CCND3, CCR2, CD151, CD180, CD1D, CD276, CD300A, CD55, CD70, CD74, CD86, CDKN1A, CEBPB, CORO1A, CR1, EPHB2, FCGR2B, FCGR3A, FYN, GAPT, GPR183, HAVCR2, HHLA2, HLA-DMB, HLA-DPA1, HLA-DPB1, HLA-DRB1, HLA-E, IGFBP2, IL10, IL12RB1, IL15, IL6ST, IRS2, JAK2, LEP, LGALS3, LILRB1, LILRB2, LILRB4, LST1, LYN, MNDA, MPL, MYD88, MZB1, NDFIP1, NFATC2, P2RX7, PELI1, PLCL2, PRKCD, PRKCQ, PRNP, PTPN6, PTPRC, PYCARD, SASH3, SDC4, SLAMF1, SLC11A1, SLC7A1, SPN, TGFBR2, TLR4, TNFRSF1B, TNFRSF4, TNFSF13B, TNFSF14, TNFSF8, TNFSF9, TYROBP, VSIG4, ZBTB7B |
| GO:0050670 | regulation of lymphocyte proliferation | 73 | 3.96E-21 | 2.86E-21 | ADA, AHR, AIF1, BCL2, BCL6, BST1, BTN2A2, CASP3, CCDC88B, CCR2, CD1D, CD276, CD300A, CD55, CD70, CD74, CD86, CDKN1A, CEBPB, CORO1A, CR1, EPHB2, FCGR2B, FCGR3A, GPR183, HAVCR2, HHLA2, HLA-DMB, HLA-DPA1, HLA-DPB1, HLA-DRB1, HLA-E, IGFBP2, IL10, IL12RB1, IL15, IL6ST, IRS2, JAK2, LEP, LGALS3, LILRB1, LILRB2, LILRB4, LST1, LYN, MNDA, MPL, MYD88, MZB1, NDFIP1, NFATC2, PELI1, PRKCQ, PRNP, PTPN6, PTPRC, PYCARD, SASH3, SDC4, SLAMF1, SLC7A1, SPN, TGFBR2, TLR4, TNFRSF1B, TNFRSF4, TNFSF13B, TNFSF8, TNFSF9, TYROBP, VSIG4, ZBTB7B |
| GO:0070661 | leukocyte proliferation | 90 | 3.96E-21 | 2.86E-21 | ADA, AHR, AIF1, ARMC5, AZI2, BCL2, BCL6, BST1, BST2, BTN2A2, CASP3, CCDC88B, CCND3, CCR2, CD151, CD180, CD1D, CD276, CD300A, CD55, CD70, CD74, CD86, CDKN1A, CEBPB, CORO1A, CR1, CSF1R, CSF2RA, EPHB2, FCGR2B, FCGR3A, FYN, GAPT, GPR183, HAVCR2, HHLA2, HLA-DMB, HLA-DPA1, HLA-DPB1, HLA-DRB1, HLA-E, IGFBP2, IL10, IL12RB1, IL15, IL6ST, IRS2, JAK2, LEP, LGALS3, LILRB1, LILRB2, LILRB4, LST1, LYN, MNDA, MPL, MYD88, MZB1, NDFIP1, NFATC2, P2RX7, PELI1, PLCL2, PRKCD, PRKCQ, PRNP, PTPN6, PTPRC, PYCARD, SASH3, SDC4, SLAMF1, SLC11A1, SLC7A1, SPN, TCIRG1, TGFBR2, TLR4, TNFAIP3, TNFRSF1B, TNFRSF4, TNFSF13B, TNFSF14, TNFSF8, TNFSF9, TYROBP, VSIG4, ZBTB7B |
| GO:0032943 | mononuclear cell proliferation | 85 | 4.04E-21 | 2.91E-21 | ADA, AHR, AIF1, ARMC5, AZI2, BCL2, BCL6, BST1, BTN2A2, CASP3, CCDC88B, CCND3, CCR2, CD151, CD180, CD1D, CD276, CD300A, CD55, CD70, CD74, CD86, CDKN1A, CEBPB, CORO1A, CR1, EPHB2, FCGR2B, FCGR3A, FYN, GAPT, GPR183, HAVCR2, HHLA2, HLA-DMB, HLA-DPA1, HLA-DPB1, HLA-DRB1, HLA-E, IGFBP2, IL10, IL12RB1, IL15, IL6ST, IRS2, JAK2, LEP, LGALS3, LILRB1, LILRB2, LILRB4, LST1, LYN, MNDA, MPL, MYD88, MZB1, NDFIP1, NFATC2, P2RX7, PELI1, PLCL2, PRKCD, PRKCQ, PRNP, PTPN6, PTPRC, PYCARD, SASH3, SDC4, SLAMF1, SLC11A1, SLC7A1, SPN, TGFBR2, TLR4, TNFRSF1B, TNFRSF4, TNFSF13B, TNFSF14, TNFSF8, TNFSF9, TYROBP, VSIG4, ZBTB7B |
| GO:0070663 | regulation of leukocyte proliferation | 77 | 4.86E-21 | 3.5E-21 | ADA, AHR, AIF1, BCL2, BCL6, BST1, BST2, BTN2A2, CASP3, CCDC88B, CCR2, CD1D, CD276, CD300A, CD55, CD70, CD74, CD86, CDKN1A, CEBPB, CORO1A, CR1, CSF1R, CSF2RA, EPHB2, FCGR2B, FCGR3A, GPR183, HAVCR2, HHLA2, HLA-DMB, HLA-DPA1, HLA-DPB1, HLA-DRB1, HLA-E, IGFBP2, IL10, IL12RB1, IL15, IL6ST, IRS2, JAK2, LEP, LGALS3, LILRB1, LILRB2, LILRB4, LST1, LYN, MNDA, MPL, MYD88, MZB1, NDFIP1, NFATC2, PELI1, PRKCQ, PRNP, PTPN6, PTPRC, PYCARD, SASH3, SDC4, SLAMF1, SLC7A1, SPN, TGFBR2, TLR4, TNFAIP3, TNFRSF1B, TNFRSF4, TNFSF13B, TNFSF8, TNFSF9, TYROBP, VSIG4, ZBTB7B |
| GO:0050867 | positive regulation of cell activation | 106 | 6.62E-21 | 4.78E-21 | ADA, ADAM8, AIF1, BCL2, BCL6, BST1, BTN2A2, CAPN3, CCDC88B, CCR2, CD1D, CD276, CD4, CD55, CD70, CD74, CD83, CD86, CDKN1A, CLEC4D, CLEC7A, CORO1A, CR1, EPHB2, FCGR3A, FGR, FYN, GATA2, GPR183, HAVCR2, HHLA2, HLA-DMB, HLA-DPA1, HLA-DPB1, HLA-DQA1, HLA-DQB1, HLA-DQB2, HLA-DRA, HLA-DRB1, HLA-DRB5, HLA-E, HLX, IGFBP2, IGLL1, IL10, IL12RB1, IL15, IL4R, IL6ST, IRS2, ITGAM, ITGB2, JAK2, LEP, LGALS1, LILRA2, LILRA5, LILRB1, LILRB2, LILRB4, LRRK2, LYN, MDK, MMP8, MPL, MYB, MYD88, NFATC2, NFKBIZ, NLRP3, NOD2, PELI1, PRKCQ, PTAFR, PTPN6, PTPRC, PYCARD, RARA, RHOH, RUNX1, SASH3, SIRPA, SIRPB1, SLAMF1, SLC7A1, SOX12, SOX4, SRC, TESPA1, TGFB1, TGFBR2, THBS1, TLR4, TLR6, TNFRSF4, TNFSF13B, TNFSF14, TNFSF9, TOX, TYROBP, VNN1, XBP1, YES1, ZBTB16, ZBTB7B, ZMIZ1 |
| GO:0032944 | regulation of mononuclear cell proliferation | 73 | 6.86E-21 | 4.95E-21 | ADA, AHR, AIF1, BCL2, BCL6, BST1, BTN2A2, CASP3, CCDC88B, CCR2, CD1D, CD276, CD300A, CD55, CD70, CD74, CD86, CDKN1A, CEBPB, CORO1A, CR1, EPHB2, FCGR2B, FCGR3A, GPR183, HAVCR2, HHLA2, HLA-DMB, HLA-DPA1, HLA-DPB1, HLA-DRB1, HLA-E, IGFBP2, IL10, IL12RB1, IL15, IL6ST, IRS2, JAK2, LEP, LGALS3, LILRB1, LILRB2, LILRB4, LST1, LYN, MNDA, MPL, MYD88, MZB1, NDFIP1, NFATC2, PELI1, PRKCQ, PRNP, PTPN6, PTPRC, PYCARD, SASH3, SDC4, SLAMF1, SLC7A1, SPN, TGFBR2, TLR4, TNFRSF1B, TNFRSF4, TNFSF13B, TNFSF8, TNFSF9, TYROBP, VSIG4, ZBTB7B |
| GO:0045785 | positive regulation of cell adhesion | 107 | 1.11E-20 | 7.98E-21 | ADA, ADAM8, ADAM9, AIF1, ALOX5, BCL6, BTN2A2, CALR, CCDC88B, CCR2, CD1D, CD276, CD36, CD4, CD55, CD70, CD74, CD83, CD86, CDK6, CHST2, CORO1A, CR1, EMILIN2, F11R, FLNA, FLOT1, FMN1, FSTL3, FYN, GCNT2, HAVCR2, HHLA2, HLA-DMB, HLA-DPA1, HLA-DPB1, HLA-DQA1, HLA-DQB1, HLA-DQB2, HLA-DRA, HLA-DRB1, HLA-DRB5, HLA-E, HLX, IGFBP2, IL10, IL12RB1, IL15, IL4R, IL6ST, ITGA4, ITGB2, JAK2, KIF26B, KIFAP3, LDB1, LEP, LGALS1, LILRB1, LILRB2, LILRB4, LYN, MDK, MYB, NEDD9, NFAT5, NFKBIZ, NID1, NLRP3, NOD2, PLAUR, PREX1, PRKCA, PRKCQ, PTAFR, PTPN6, PTPRC, PTPRJ, PYCARD, RARA, RHOH, RIN2, RRAS, RUNX1, S100A10, SASH3, SDC4, SIRPA, SIRPB1, SLAMF1, SLC7A1, SOX12, SOX4, SRC, ST3GAL4, STX3, TESPA1, TGFBR2, TNFSF13B, TNFSF14, TNFSF9, VNN1, XBP1, YES1, ZBTB16, ZBTB7B, ZMIZ1 |
| GO:0002696 | positive regulation of leukocyte activation | 103 | 1.42E-20 | 1.03E-20 | ADA, ADAM8, AIF1, BCL2, BCL6, BST1, BTN2A2, CCDC88B, CCR2, CD1D, CD276, CD4, CD55, CD70, CD74, CD83, CD86, CDKN1A, CLEC4D, CLEC7A, CORO1A, CR1, EPHB2, FCGR3A, FGR, FYN, GATA2, GPR183, HAVCR2, HHLA2, HLA-DMB, HLA-DPA1, HLA-DPB1, HLA-DQA1, HLA-DQB1, HLA-DQB2, HLA-DRA, HLA-DRB1, HLA-DRB5, HLA-E, HLX, IGFBP2, IGLL1, IL10, IL12RB1, IL15, IL4R, IL6ST, IRS2, ITGAM, ITGB2, JAK2, LEP, LGALS1, LILRB1, LILRB2, LILRB4, LRRK2, LYN, MDK, MMP8, MPL, MYB, MYD88, NFATC2, NFKBIZ, NLRP3, NOD2, PELI1, PRKCQ, PTAFR, PTPN6, PTPRC, PYCARD, RARA, RHOH, RUNX1, SASH3, SIRPA, SIRPB1, SLAMF1, SLC7A1, SOX12, SOX4, SRC, TESPA1, TGFB1, TGFBR2, THBS1, TLR4, TLR6, TNFRSF4, TNFSF13B, TNFSF14, TNFSF9, TOX, TYROBP, VNN1, XBP1, YES1, ZBTB16, ZBTB7B, ZMIZ1 |
| GO:1902105 | regulation of leukocyte differentiation | 81 | 1.56E-20 | 1.12E-20 | ADA, ADAM8, BCL6, BTN2A2, CCR1, CCR2, CD4, CD74, CD83, CD86, CDK6, CEBPB, CR1, EVI2B, FCGR2B, FGL2, FOS, FSTL3, GATA2, GPR137B, HLA-B, HLA-DRA, HLA-DRB1, HLX, ID2, IL10, IL12RB1, IL15, IL4R, IRF7, LILRB1, LILRB2, LILRB3, LILRB4, LOXL3, LYN, MAFB, MDK, MITF, MYB, MYC, NDFIP1, NFAM1, NFKBIZ, NLRP3, NOTCH2, PPARGC1B, PRKCA, PTPN6, PTPRC, RARA, RASSF2, RB1, RHOH, RUNX1, SASH3, SLC46A2, SLC9B2, SOX12, SOX4, TESPA1, TGFB1, TGFBR2, TLR4, TMEM176A, TMEM176B, TMEM64, TNFAIP6, TNFSF9, TOX, TRIB1, TYROBP, VNN1, XBP1, ZBTB16, ZBTB46, ZBTB7B, ZEB1, ZFP36L1, ZFPM1, ZMIZ1 |
| GO:0006909 | phagocytosis | 84 | 1.23E-19 | 8.84E-20 | ABCA7, AIF1, APOA2, ARHGAP12, ATG3, BCR, BIN2, C2, C3, CALR, CCR2, CD14, CD300A, CD300LF, CD302, CD36, CD93, CDC42SE1, CEACAM4, CLEC7A, CORO1A, CYBA, DYSF, FCER1G, FCGR1A, FCGR2B, FCN1, FGR, FPR2, FYN, GATA2, HCK, IGLL1, IL15, IL2RG, IRF8, ITGAL, ITGAM, ITGB2, LDLR, LEP, LRP1, LYN, LYST, MARCO, MFGE8, MSR1, MYD88, MYO18A, MYO1G, MYO7A, NCF2, NOD2, P2RX7, P2RY6, PAK1, PECAM1, PLA2G6, PLD2, PRKCD, PTPRC, PTPRJ, PTX3, PYCARD, RAB27A, RAB31, RAB39A, RAB7B, RARA, RHOBTB1, RHOH, SCARB1, SIRPA, SIRPB1, SLAMF1, SLC11A1, SRC, SYT11, THBS1, TLR2, TLR4, TYROBP, VAV2, YES1 |
| **CC terms** |  |  |  |  |  |
| GO:0030667 | secretory granule membrane | 94 | 3.43E-26 | 2.7E-26 | ADAM8, ALDH3B1, APLP2, ATP6V0A1, BRI3, BST1, BST2, C3AR1, C5AR1, CD14, CD300A, CD36, CD53, CD55, CD59, CD63, CD93, CEACAM3, CKAP4, CLEC12A, CLEC4C, CLEC4D, CPNE3, CR1, CXCR1, CXCR2, CYBA, CYBB, CYSTM1, FCAR, FCER1G, FCGR2A, FCGR3B, FPR1, FPR2, GAA, GLIPR1, GPR84, HLA-B, HLA-C, HVCN1, IGF2R, IQGAP1, ITGAL, ITGAM, ITGAX, ITGB2, ITPR2, LAIR1, LGALS3, LILRB2, LILRB3, LPCAT1, MCEMP1, MFGE8, MGAM, MMP25, MOSPD2, NBEAL2, NFAM1, OLR1, PECAM1, PLAU, PLAUR, PRCP, PSAP, PTAFR, PTPRC, PTPRJ, RAB27A, RAB31, RAB37, RAB3D, SERPINB10, SIGLEC14, SIGLEC9, SIRPA, SIRPB1, SLC11A1, SLC17A9, SLC27A2, SLC2A3, SLCO4C1, STX3, STXBP5, SVIP, TCIRG1, TLR2, TNFRSF1B, TOM1, TRPM2, TSPAN14, TYROBP, VNN1 |
| GO:0070820 | tertiary granule | 66 | 3.43E-26 | 2.7E-26 | ADAM8, ASAH1, ATP6V0A1, CAMP, CD300A, CD53, CD55, CD59, CD93, CDA, CFP, CLEC12A, CLEC4C, CLEC4D, CR1, CST3, CSTB, CTSD, CTSH, CTSS, CXCL1, CYBA, CYBB, CYSTM1, FCAR, FCER1G, FPR1, FPR2, FTH1, GAA, GPR84, HP, IDH1, ITGAM, ITGAX, ITGB2, LAIR1, LGALS3, LILRB2, LTA4H, LYZ, MCEMP1, METTL7A, MGAM, MMP8, NBEAL2, OLR1, OSCAR, PLAU, PRCP, PTAFR, PTPN6, PTX3, QPCT, SERPINB10, SIGLEC14, SIRPA, SLC11A1, SLC2A3, STXBP2, SVIP, TCIRG1, TIMP2, TNFAIP6, TRPM2, TSPAN14 |
| GO:0101002 | ficolin-1-rich granule | 66 | 8.23E-23 | 6.47E-23 | ADAM8, ALOX5, APAF1, ARPC5, ASAH1, ATG7, ATP6V0A1, BIN2, CD300A, CD55, CD93, CDA, CLEC4C, CLEC4D, COMMD9, COTL1, CR1, CRISPLD2, CST3, CSTB, CTSB, CTSD, CTSH, CTSS, CTSZ, FCAR, FCER1G, FCN1, FGL2, FPR1, FPR2, FTH1, GAA, GNS, GUSB, HK3, HSPA1A, HSPA6, IDH1, IMPDH1, ITGAX, ITGB2, LGALS3, LILRB2, LTA4H, MGAM, MNDA, MVP, NBEAL2, OSTF1, PGAM1, PKM, PRCP, PYGL, QPCT, SERPINA1, SERPINB10, SIGLEC14, SIRPA, SLC11A1, SLC2A3, TCIRG1, TIMP2, TNFAIP6, TRPM2, VCL |
| GO:0042581 | specific granule | 57 | 1.18E-19 | 9.24E-20 | ADAM8, ALDH3B1, BPI, BST1, C3AR1, CAMP, CD36, CD53, CD59, CD93, CEACAM3, CFP, CKAP4, CLEC12A, CLEC4D, CTSD, CTSZ, CXCL1, CYBA, CYBB, FCAR, FPR2, GPR84, HP, HPSE, HVCN1, ITGAL, ITGAM, ITGB2, LAIR1, LYZ, MCEMP1, MMP25, MMP8, MOSPD2, OLR1, OSCAR, PDXK, PLAU, PLAUR, PTPN6, PTPRJ, PTX3, QPCT, RAB27A, RAB37, SLC27A2, SLC2A3, SLCO4C1, STX3, STXBP2, TIMP2, TNFRSF1B, TOM1, TRPM2, TSPAN14, VCL |
| GO:0070821 | tertiary granule membrane | 34 | 9.75E-16 | 7.66E-16 | ADAM8, CD300A, CD53, CD59, CD93, CLEC12A, CLEC4C, CLEC4D, CYBA, CYBB, CYSTM1, FCAR, FCER1G, FPR2, GAA, GPR84, ITGAM, ITGAX, ITGB2, LAIR1, LILRB2, MCEMP1, MGAM, NBEAL2, OLR1, PLAU, PTAFR, SIGLEC14, SIRPA, SLC11A1, SLC2A3, SVIP, TRPM2, TSPAN14 |
| GO:0060205 | cytoplasmic vesicle lumen | 77 | 6.37E-15 | 5.01E-15 | ADA, ALOX5, ANXA2, APAF1, ARPC5, ARSA, ATG7, BACE1, BIN2, BPI, C3, CAMP, CDA, CFP, COMMD9, COTL1, CPPED1, CRISPLD2, CSTB, CTSD, CTSH, CTSW, CTSZ, CXCL1, F5, FCN1, FGR, FTL, FUCA1, GCA, GM2A, GNS, GRN, GUSB, HEXB, HK3, HP, HPSE, HSPA6, IDH1, IMPDH1, LYZ, MAN2B1, MMP8, MNDA, MVP, NPC2, OSCAR, OSTF1, PADI2, PDXK, PGAM1, PKM, PRKCD, PRSS57, PTPN6, PTX3, PYCARD, PYGL, QPCT, RAB27A, RNASE2, S100A11, S100A12, S100A8, S100A9, SDCBP, SERPINA1, SERPINE1, SRGN, TGFB1, THBS1, TIMP1, TIMP2, TMSB4X, VCL, VPS13A |
| GO:0035579 | specific granule membrane | 37 | 6.5E-15 | 5.11E-15 | ADAM8, ALDH3B1, BST1, C3AR1, CD36, CD53, CD59, CD93, CEACAM3, CKAP4, CLEC12A, CLEC4D, CYBA, CYBB, FCAR, FPR2, GPR84, HVCN1, ITGAL, ITGAM, ITGB2, LAIR1, MCEMP1, MMP25, MOSPD2, OLR1, PLAU, PLAUR, PTPRJ, RAB37, SLC27A2, SLC2A3, SLCO4C1, TNFRSF1B, TOM1, TRPM2, TSPAN14 |
| GO:0031983 | vesicle lumen | 77 | 6.91E-15 | 5.43E-15 | ADA, ALOX5, ANXA2, APAF1, ARPC5, ARSA, ATG7, BACE1, BIN2, BPI, C3, CAMP, CDA, CFP, COMMD9, COTL1, CPPED1, CRISPLD2, CSTB, CTSD, CTSH, CTSW, CTSZ, CXCL1, F5, FCN1, FGR, FTL, FUCA1, GCA, GM2A, GNS, GRN, GUSB, HEXB, HK3, HP, HPSE, HSPA6, IDH1, IMPDH1, LYZ, MAN2B1, MMP8, MNDA, MVP, NPC2, OSCAR, OSTF1, PADI2, PDXK, PGAM1, PKM, PRKCD, PRSS57, PTPN6, PTX3, PYCARD, PYGL, QPCT, RAB27A, RNASE2, S100A11, S100A12, S100A8, S100A9, SDCBP, SERPINA1, SERPINE1, SRGN, TGFB1, THBS1, TIMP1, TIMP2, TMSB4X, VCL, VPS13A |
| GO:0009897 | external side of plasma membrane | 95 | 7.46E-15 | 5.87E-15 | ABCG1, ADA, ADAM9, ANXA5, ASGR2, BMPR1A, BTN2A2, CALR, CAPN2, CCR1, CCR2, CCR5, CCRL2, CD14, CD163, CD163L1, CD1C, CD1D, CD1E, CD200R1, CD276, CD302, CD36, CD4, CD48, CD59, CD69, CD74, CD83, CD86, CLEC10A, CLEC12B, CLEC2B, CLEC2D, CLEC4C, CLEC4D, CLEC4E, CLEC7A, CSF2RA, CTSB, CX3CR1, CXCL10, CXCR1, CXCR2, CXCR4, FCER1G, FCER2, FCGR2B, FCGR3A, FCGRT, FCN1, FLOT1, FOLR2, HFE, HHLA2, HLA-DRB1, HLA-E, ICAM1, IGLL1, IL12RB1, IL13RA1, IL2RG, IL31RA, IL4R, IL6R, IL6ST, ITGA4, ITGA6, ITGA7, ITGAL, ITGAM, ITGAX, ITGB2, LDLR, LILRB1, MFGE8, MPL, MSR1, P2RX7, PRLR, PRNP, PTPRC, RTN4R, SCUBE1, SLAMF1, SLC4A3, SLC7A5, SPN, TGFBR2, THBD, THBS1, TLR4, TLR8, TNFRSF11A, TNFRSF4 |
| GO:0030139 | endocytic vesicle | 78 | 2.38E-14 | 1.87E-14 | ADAM8, AMN, ARRB2, ATP6V0A1, ATP6V0D1, ATP6V0E2, CALR, CAMK2D, CD163, CD36, CD4, CD74, CLEC4E, CLTCL1, CORO1A, CTSL, CTSS, CYBA, CYBB, DYSF, EHD4, FCGR1A, FZD2, FZD5, HBEGF, HLA-B, HLA-C, HLA-DPA1, HLA-DPB1, HLA-DQA1, HLA-DQB1, HLA-DQB2, HLA-DRA, HLA-DRB1, HLA-DRB5, HLA-E, HP, HVCN1, IGF2R, IRGM, ITGB5, LDLR, LPAR1, LRP1, LYN, MARCO, MDM2, MPEG1, MSR1, MTSS1, MYO1C, MYO1E, NCF1, NCF2, NOD2, RAB11FIP1, RAB11FIP5, RAB31, RAB38, RAB39A, RAB7B, RIN1, RIN2, SCARB1, SCIMP, SLAMF1, SLC11A1, SMO, SRGAP2, SYT11, TCIRG1, TLR1, TLR2, TLR6, TLR7, UNC93B1, VIM, ZYX |
| GO:0034774 | secretory granule lumen | 75 | 2.39E-14 | 1.88E-14 | ALOX5, ANXA2, APAF1, ARPC5, ARSA, ATG7, BIN2, BPI, C3, CAMP, CDA, CFP, COMMD9, COTL1, CPPED1, CRISPLD2, CSTB, CTSD, CTSH, CTSW, CTSZ, CXCL1, F5, FCN1, FGR, FTL, FUCA1, GCA, GM2A, GNS, GRN, GUSB, HEXB, HK3, HP, HPSE, HSPA6, IDH1, IMPDH1, LYZ, MAN2B1, MMP8, MNDA, MVP, NPC2, OSCAR, OSTF1, PADI2, PDXK, PGAM1, PKM, PRKCD, PRSS57, PTPN6, PTX3, PYCARD, PYGL, QPCT, RAB27A, RNASE2, S100A11, S100A12, S100A8, S100A9, SDCBP, SERPINA1, SERPINE1, SRGN, TGFB1, THBS1, TIMP1, TIMP2, TMSB4X, VCL, VPS13A |
| GO:0005925 | focal adhesion | 87 | 1.58E-13 | 1.24E-13 | ADAM9, AHNAK, AIF1L, ANXA5, ANXA6, ARHGAP24, ARHGAP26, ARHGAP31, ARMC5, ARPC5, CALR, CAPN2, CBL, CD151, CD59, CD99, CDC42EP1, CPNE3, CSPG4, CSRP1, CTNNA1, CYBA, DOCK7, DST, FGFR3, FLNA, FLOT1, FZD1, FZD2, HCK, HSPA1A, HSPG2, ICAM1, IGF2R, IQGAP1, ITGA4, ITGA6, ITGB2, ITGB5, ITGB7, JAK2, KLF11, LCP1, LPXN, LRP1, MARCKS, MPRIP, MPZL1, MRC2, NEDD9, NEXN, NHS, PAK1, PALLD, PLAU, PLAUR, PLEC, PPFIBP1, PTK7, PTPN12, PTPRC, RHOU, RPL10A, RPL22, RPL3, RPL37A, RPL9, RPS4X, RRAS, SDC4, SDCBP, SLA, SNTB2, SORBS3, SRC, STARD8, TLN1, TNFSF13B, TNS3, TPM4, TRIP6, VASP, VCL, VIM, WASF1, YES1, ZYX |
| GO:0005775 | vacuolar lumen | 50 | 2.75E-13 | 2.17E-13 | ANXA2, ARSA, ASAH1, BPI, C3, CD1E, CD74, CPPED1, CSPG4, CTSB, CTSD, CTSF, CTSL, CTSS, DAPK2, EPDR1, FTL, FUCA1, GAA, GCA, GM2A, GNS, GRN, GUSB, HEXB, HPSE, HSPG2, IFI30, IMPDH1, LIPA, LYZ, MAN2B1, MNDA, NAAA, NPC2, PADI2, PLBD2, PLD3, PPT1, PRKCD, PRSS57, PSAP, PYCARD, RNASE2, SDC2, SDC4, SDCBP, SGSH, TCN2, VCAN |
| GO:0030055 | cell-substrate junction | 87 | 5.01E-13 | 3.94E-13 | ADAM9, AHNAK, AIF1L, ANXA5, ANXA6, ARHGAP24, ARHGAP26, ARHGAP31, ARMC5, ARPC5, CALR, CAPN2, CBL, CD151, CD59, CD99, CDC42EP1, CPNE3, CSPG4, CSRP1, CTNNA1, CYBA, DOCK7, DST, FGFR3, FLNA, FLOT1, FZD1, FZD2, HCK, HSPA1A, HSPG2, ICAM1, IGF2R, IQGAP1, ITGA4, ITGA6, ITGB2, ITGB5, ITGB7, JAK2, KLF11, LCP1, LPXN, LRP1, MARCKS, MPRIP, MPZL1, MRC2, NEDD9, NEXN, NHS, PAK1, PALLD, PLAU, PLAUR, PLEC, PPFIBP1, PTK7, PTPN12, PTPRC, RHOU, RPL10A, RPL22, RPL3, RPL37A, RPL9, RPS4X, RRAS, SDC4, SDCBP, SLA, SNTB2, SORBS3, SRC, STARD8, TLN1, TNFSF13B, TNS3, TPM4, TRIP6, VASP, VCL, VIM, WASF1, YES1, ZYX |
| GO:0045121 | membrane raft | 72 | 1.25E-12 | 9.82E-13 | AHNAK, ANXA2, ARID3A, ATP1B1, BACE1, BIRC3, BMPR1A, BST2, BVES, CAPN2, CASP3, CBL, CD14, CD36, CD4, CD48, CD55, CR1, CTNNA1, CTSD, EFHD2, F2R, FLOT1, FYN, GABBR1, HCK, HK1, HMOX1, HPSE, ICAM1, IGF1R, IL6ST, ITGAM, ITGB2, JAK2, KCNE1, KCNE3, KCNQ1, LRRK2, LYN, MLC1, MS4A4A, MYO1C, MYOF, NFAM1, OLR1, ORAI1, PAG1, PECAM1, PI4K2A, PPT1, PRNP, PTGS2, PTPRC, RGS19, RTN4R, S100A10, SCARB1, SDC4, SDCBP, SELPLG, SMO, SPRED1, SRC, SYNJ2, TFPI, TGFBR2, TLR1, TLR2, TLR6, TNFRSF1B, UNC5A |
| GO:0098857 | membrane microdomain | 72 | 1.38E-12 | 1.08E-12 | AHNAK, ANXA2, ARID3A, ATP1B1, BACE1, BIRC3, BMPR1A, BST2, BVES, CAPN2, CASP3, CBL, CD14, CD36, CD4, CD48, CD55, CR1, CTNNA1, CTSD, EFHD2, F2R, FLOT1, FYN, GABBR1, HCK, HK1, HMOX1, HPSE, ICAM1, IGF1R, IL6ST, ITGAM, ITGB2, JAK2, KCNE1, KCNE3, KCNQ1, LRRK2, LYN, MLC1, MS4A4A, MYO1C, MYOF, NFAM1, OLR1, ORAI1, PAG1, PECAM1, PI4K2A, PPT1, PRNP, PTGS2, PTPRC, RGS19, RTN4R, S100A10, SCARB1, SDC4, SDCBP, SELPLG, SMO, SPRED1, SRC, SYNJ2, TFPI, TGFBR2, TLR1, TLR2, TLR6, TNFRSF1B, UNC5A |
| GO:0101003 | ficolin-1-rich granule membrane | 27 | 3.28E-12 | 2.58E-12 | ADAM8, ATP6V0A1, CD300A, CD55, CD93, CLEC4C, CLEC4D, CR1, FCAR, FCER1G, FPR1, FPR2, GAA, ITGAX, ITGB2, LGALS3, LILRB2, MGAM, NBEAL2, PRCP, SERPINB10, SIGLEC14, SIRPA, SLC11A1, SLC2A3, TCIRG1, TRPM2 |
| GO:1904813 | ficolin-1-rich granule lumen | 39 | 7.85E-12 | 6.17E-12 | ALOX5, APAF1, ARPC5, ASAH1, ATG7, BIN2, CDA, COMMD9, COTL1, CRISPLD2, CST3, CSTB, CTSB, CTSD, CTSH, CTSS, CTSZ, FCN1, FGL2, FTH1, GNS, GUSB, HK3, HSPA1A, HSPA6, IDH1, IMPDH1, LTA4H, MNDA, MVP, OSTF1, PGAM1, PKM, PYGL, QPCT, SERPINA1, TIMP2, TNFAIP6, VCL |
| GO:0005766 | primary lysosome | 44 | 1.21E-11 | 9.49E-12 | ANXA2, ARSA, BPI, BRI3, BST2, C3, C3AR1, CD63, CKAP4, CPNE3, CPPED1, FPR1, FTL, FUCA1, GAA, GCA, GLIPR1, GM2A, GNS, GRN, GUSB, HEXB, IMPDH1, LPCAT1, LYZ, MAN2B1, MNDA, NFAM1, NPC2, PADI2, PRCP, PRKCD, PRSS57, PSAP, PYCARD, RAB37, RAB3D, RNASE2, SDCBP, SLCO4C1, STX3, STXBP2, TOM1, VNN1 |
| GO:0042582 | azurophil granule | 44 | 1.21E-11 | 9.49E-12 | ANXA2, ARSA, BPI, BRI3, BST2, C3, C3AR1, CD63, CKAP4, CPNE3, CPPED1, FPR1, FTL, FUCA1, GAA, GCA, GLIPR1, GM2A, GNS, GRN, GUSB, HEXB, IMPDH1, LPCAT1, LYZ, MAN2B1, MNDA, NFAM1, NPC2, PADI2, PRCP, PRKCD, PRSS57, PSAP, PYCARD, RAB37, RAB3D, RNASE2, SDCBP, SLCO4C1, STX3, STXBP2, TOM1, VNN1 |
| **MF terms** |  |  |  |  |  |
| GO:0140375 | immune receptor activity | 54 | 3.4E-17 | 2.82E-17 | C3AR1, C5AR1, C5AR2, CCR1, CCR2, CCR5, CCRL2, CD200R1, CD4, CD74, CMKLR1, CR1, CSF2RA, CTSH, CX3CR1, CXCR1, CXCR2, CXCR4, FCER1G, FCGR1A, FCGR2B, FCGR3A, FPR1, FPR2, GPR35, HLA-DPA1, HLA-DQA1, HLA-DQB1, HLA-DQB2, HLA-DRA, HLA-DRB1, IFNGR1, IFNGR2, IL10RA, IL12RB1, IL13RA1, IL17RA, IL17RE, IL1R2, IL2RG, IL31RA, IL4R, IL6R, IL6ST, LILRA1, LILRA2, LILRA4, LILRA5, LILRA6, LILRB1, LILRB2, LILRB3, MPL, PRLR |
| GO:0003779 | actin binding | 81 | 1.49E-07 | 1.24E-07 | ABI3, ADD3, AIF1, AIF1L, ANXA6, ARPC5, CACNB2, CAPG, CCR5, CORO1A, COTL1, CTNNA1, CXCR4, DBN1, DIAPH2, DST, ENC1, EPB41L3, EPS8, ERMN, FGD4, FKBP15, FLNA, FMN1, FMNL2, FSCN1, GAS7, HIP1, HIP1R, IQGAP1, JMY, KLHL2, LCP1, LRRK2, LSP1, MACF1, MAP1A, MARCKS, MEFV, MICAL1, MICAL2, MICAL3, MPRIP, MSRB1, MTSS1, MYO18A, MYO1B, MYO1C, MYO1E, MYO1F, MYO1G, MYO5A, MYO5C, MYO7A, MYOM2, MYOZ3, NEXN, NOD2, PALLD, PDLIM5, PHACTR1, PHACTR3, PLEC, PPP1R18, PSTPIP1, S100A4, SNTB2, SPTBN1, SPTBN2, SSH2, TLN1, TMSB10, TMSB4X, TNNI2, TPM2, TPM4, VASH1, VASH2, VASP, VCL, WASF1 |
| GO:0038187 | pattern recognition receptor activity | 15 | 2E-07 | 1.66E-07 | CD14, CD36, CLEC4D, CLEC4E, CLEC7A, FCN1, LY96, MARCO, NOD2, PTAFR, SCARB1, TLR2, TLR4, TLR7, TLR8 |
| GO:0030695 | GTPase regulator activity | 83 | 1.97E-06 | 1.64E-06 | ABR, ADAP1, ADAP2, ADPRH, AGAP3, ALS2, ANKRD27, ARAP1, ARAP3, ARHGAP12, ARHGAP18, ARHGAP23, ARHGAP24, ARHGAP26, ARHGAP27, ARHGAP31, ARHGAP5, ARHGAP6, ARHGEF10L, ARHGEF11, ARHGEF17, ARHGEF3, ARHGEF40, ASAP1, BCR, C9orf72, CDC42SE1, CPEB2, CYTH4, DENND1A, DNMBP, DOCK10, DOCK3, DOCK4, DOCK7, EVI5, FGD2, FGD4, FGD6, GPSM1, GPSM3, HPS4, IQGAP1, IQSEC1, IQSEC2, IQSEC3, ITSN1, LRRK2, NET1, NUCB2, OBSCN, PLEKHG4, PLXNB1, PREX1, RALGPS2, RASGEF1B, RASGRP4, RCBTB2, RGS14, RGS2, RGS3, RHOH, RHOU, RIN1, RIN2, RP2, RTKN, SBF2, SH3BP5, SIPA1, SIRPA, SMAP2, SRGAP2, STARD8, STXBP5, SYNGAP1, TAGAP, TBC1D12, TBC1D16, TBC1D2, TBC1D8, TBC1D9, VAV2 |
| GO:0060589 | nucleoside-triphosphatase regulator activity | 83 | 1.97E-06 | 1.64E-06 | ABR, ADAP1, ADAP2, ADPRH, AGAP3, ALS2, ANKRD27, ARAP1, ARAP3, ARHGAP12, ARHGAP18, ARHGAP23, ARHGAP24, ARHGAP26, ARHGAP27, ARHGAP31, ARHGAP5, ARHGAP6, ARHGEF10L, ARHGEF11, ARHGEF17, ARHGEF3, ARHGEF40, ASAP1, BCR, C9orf72, CDC42SE1, CPEB2, CYTH4, DENND1A, DNMBP, DOCK10, DOCK3, DOCK4, DOCK7, EVI5, FGD2, FGD4, FGD6, GPSM1, GPSM3, HPS4, IQGAP1, IQSEC1, IQSEC2, IQSEC3, ITSN1, LRRK2, NET1, NUCB2, OBSCN, PLEKHG4, PLXNB1, PREX1, RALGPS2, RASGEF1B, RASGRP4, RCBTB2, RGS14, RGS2, RGS3, RHOH, RHOU, RIN1, RIN2, RP2, RTKN, SBF2, SH3BP5, SIPA1, SIRPA, SMAP2, SRGAP2, STARD8, STXBP5, SYNGAP1, TAGAP, TBC1D12, TBC1D16, TBC1D2, TBC1D8, TBC1D9, VAV2 |
| GO:0005543 | phospholipid binding | 80 | 2.2E-06 | 1.82E-06 | ABCG1, ADAP1, ADAP2, ANXA2, ANXA5, ANXA6, APOA2, ARAP1, ARAP3, ARHGAP26, ASAP1, BIN2, CAPG, CD300A, CD300LF, CPNE3, DAPP1, DENND1A, DOC2A, DYSF, EPDR1, ESYT3, FCGR3B, FGD2, GGA2, GSDMB, HIP1, HIP1R, IQGAP1, ITPR2, KCNJ1, KCNJ2, KCNQ1, KIF16B, LAPTM4B, LPAR1, MFGE8, MTSS1, MYO1B, MYO1E, MYO1G, MYOF, NCF1, NRGN, OBSCN, OGT, PIK3C2B, PITPNC1, PITPNM1, PITPNM2, PLA2G2C, PLA2G4C, PLA2G7, PLD2, PLEKHA5, PLTP, PPT1, PREX1, PSAP, PTAFR, RPH3A, SBF2, SCARB1, SDCBP, SH3PXD2A, SNX18, SNX24, SNX25, SNX30, SPTBN1, SPTBN2, SYT11, SYT17, SYTL3, TEC, THBS1, TLN1, TOM1, WDFY1, WIPI1 |
| GO:0004896 | cytokine receptor activity | 28 | 2.46E-06 | 2.04E-06 | CCR1, CCR2, CCR5, CCRL2, CD4, CD74, CMKLR1, CSF2RA, CX3CR1, CXCR1, CXCR2, CXCR4, GPR35, IFNGR1, IFNGR2, IL10RA, IL12RB1, IL13RA1, IL17RA, IL17RE, IL1R2, IL2RG, IL31RA, IL4R, IL6R, IL6ST, MPL, PRLR |
| GO:0035325 | Toll-like receptor binding | 9 | 8.52E-06 | 7.07E-06 | CD36, LY96, MYD88, S100A8, S100A9, TLR1, TLR2, TLR6, UNC93B1 |
| GO:0033218 | amide binding | 69 | 1.36E-05 | 1.13E-05 | ACOT11, AVPR1B, BACE1, CALR, CD14, CD1C, CD1D, CD1E, CD300LF, CD36, CD74, CMKLR1, CST3, DBI, EPDR1, EPHB2, FASN, FCGR2B, FKBP4, FKBP5, FOLR2, FPR2, FZD5, GNRHR, GSAP, GSTM2, HLA-B, HLA-C, HLA-DPA1, HLA-DPB1, HLA-DQA1, HLA-DQB1, HLA-DRA, HLA-DRB1, HLA-DRB5, HLA-E, HSPG2, IGF1R, ITGAM, ITGB2, LAPTM4B, LDLR, LILRB2, LRP1, MARCO, MGST2, MSR1, NMUR1, NOD2, NPR2, PCSK5, PLTP, PPARG, PRLR, PRNP, PSAP, PTGDR2, RTN4R, RXRA, SCARB1, SLC7A5, SOAT1, SRD5A1, SSTR2, TLR1, TLR2, TLR4, TLR6, VIPR1 |
| GO:0023026 | MHC class II protein complex binding | 13 | 1.52E-05 | 1.27E-05 | ATP1B1, CD4, CD74, HLA-DMB, HLA-DPA1, HLA-DPB1, HLA-DQA1, HLA-DQB1, HLA-DQB2, HLA-DRA, HLA-DRB1, HLA-DRB5, PKM |
| GO:0023023 | MHC protein complex binding | 15 | 1.65E-05 | 1.37E-05 | ATP1B1, CD4, CD74, HLA-DMB, HLA-DPA1, HLA-DPB1, HLA-DQA1, HLA-DQB1, HLA-DQB2, HLA-DRA, HLA-DRB1, HLA-DRB5, LILRB1, LILRB2, PKM |
| GO:0019955 | cytokine binding | 33 | 2.09E-05 | 1.74E-05 | BMPR1A, CCR1, CCR2, CCR5, CCRL2, CD36, CD4, CD74, CSF1R, CSF2RA, CX3CR1, CXCR1, CXCR2, CXCR4, IFNGR1, IL10RA, IL12RB1, IL13RA1, IL1R2, IL1RN, IL2RG, IL31RA, IL6R, IL6ST, ITGA4, PLP2, PRLR, SCUBE3, TGFBR2, THBS1, TNFRSF11A, TNFRSF1B, ZFP36 |
| GO:0030228 | lipoprotein particle receptor activity | 10 | 6.1E-05 | 5.06E-05 | APOBR, CD36, CXCL16, LDLR, LRP1, LRP10, LRP12, LRP1B, OLR1, SCARB1 |
| GO:0051015 | actin filament binding | 42 | 7.61E-05 | 6.32E-05 | ABI3, ADD3, AIF1, AIF1L, ANXA6, ARPC5, CACNB2, CAPG, CORO1A, COTL1, CTNNA1, DBN1, ERMN, FLNA, FMNL2, FSCN1, GAS7, HIP1, HIP1R, IQGAP1, LCP1, MACF1, MARCKS, MICAL1, MPRIP, MYO18A, MYO1B, MYO1C, MYO1E, MYO1F, MYO1G, MYO5A, MYO5C, MYO7A, MYOM2, NEXN, PSTPIP1, SPTBN1, SPTBN2, TLN1, TPM2, TPM4 |
| GO:0038024 | cargo receptor activity | 22 | 7.61E-05 | 6.32E-05 | ABCA7, AMN, APOBR, ASGR2, CD163, CD163L1, CD36, CXCL16, FOLR2, FPR2, ITGAM, ITGB2, LDLR, LOXL3, LRP1, LRP10, LRP12, LRP1B, MARCO, MSR1, OLR1, SCARB1 |
| GO:0032396 | inhibitory MHC class I receptor activity | 8 | 9.89E-05 | 8.21E-05 | LILRA1, LILRA2, LILRA4, LILRA5, LILRA6, LILRB1, LILRB2, LILRB3 |
| GO:0001540 | amyloid-beta binding | 22 | 0.000107 | 8.86E-05 | BACE1, CD36, CD74, CST3, EPHB2, FCGR2B, FPR2, FZD5, GSAP, HSPG2, ITGAM, ITGB2, LDLR, LILRB2, LRP1, MARCO, MSR1, PRNP, SCARB1, TLR2, TLR4, TLR6 |
| GO:0035091 | phosphatidylinositol binding | 49 | 0.000125 | 0.000103 | ADAP1, ADAP2, ANXA2, ARAP1, ARAP3, ASAP1, CAPG, DAPP1, DENND1A, ESYT3, FCGR3B, FGD2, GGA2, GSDMB, HIP1, HIP1R, IQGAP1, ITPR2, KCNJ1, KCNJ2, KCNQ1, KIF16B, LAPTM4B, MYO1B, MYO1E, MYO1G, NCF1, NRGN, OBSCN, OGT, PIK3C2B, PITPNC1, PITPNM1, PITPNM2, PLD2, PLEKHA5, RPH3A, SBF2, SCARB1, SDCBP, SH3PXD2A, SNX18, SNX24, SNX25, SNX30, TLN1, TOM1, WDFY1, WIPI1 |
| GO:0019865 | immunoglobulin binding | 11 | 0.000135 | 0.000112 | FCAR, FCER1G, FCER2, FCGR1A, FCGR2A, FCGR2B, FCGR3A, FCGR3B, FCGRT, LGALS3, LILRA2 |
| GO:0042277 | peptide binding | 55 | 0.000135 | 0.000112 | AVPR1B, BACE1, CALR, CD14, CD1C, CD1D, CD1E, CD36, CD74, CMKLR1, CST3, EPHB2, FCGR2B, FPR2, FZD5, GNRHR, GSAP, GSTM2, HLA-B, HLA-C, HLA-DPA1, HLA-DPB1, HLA-DQA1, HLA-DQB1, HLA-DRA, HLA-DRB1, HLA-DRB5, HLA-E, HSPG2, IGF1R, ITGAM, ITGB2, LDLR, LILRB2, LRP1, MARCO, MGST2, MSR1, NMUR1, NOD2, NPR2, PCSK5, PPARG, PRLR, PRNP, PTGDR2, RXRA, SCARB1, SLC7A5, SSTR2, TLR1, TLR2, TLR4, TLR6, VIPR1 |
| **KEGG** |  |  |  |  |  |
| hsa05140 | Leishmaniasis | 34 | 7.27E-12 | 5.54E-12 | C3, CR1, CYBA, CYBB, FCGR1A, FCGR2A, FCGR3A, FCGR3B, FOS, HLA-DMB, HLA-DPA1, HLA-DPB1, HLA-DQA1, HLA-DQB1, HLA-DRA, HLA-DRB1, HLA-DRB5, IFNGR1, IFNGR2, IL10, ITGA4, ITGAM, ITGB2, JAK2, MAPK12, MYD88, NCF1, NCF2, PRKCB, PTGS2, PTPN6, TGFB1, TLR2, TLR4 |
| hsa05152 | Tuberculosis | 55 | 8.15E-12 | 6.2E-12 | APAF1, ATP6V0A1, ATP6V0D1, BCL2, C3, CAMK2D, CAMP, CARD9, CASP3, CD14, CD74, CEBPB, CLEC4E, CLEC7A, CORO1A, CR1, CTSD, CTSS, FCER1G, FCGR1A, FCGR2A, FCGR2B, FCGR3A, FCGR3B, HLA-DMB, HLA-DPA1, HLA-DPB1, HLA-DQA1, HLA-DQB1, HLA-DRA, HLA-DRB1, HLA-DRB5, IFNGR1, IFNGR2, IL10, IL10RA, ITGAM, ITGAX, ITGB2, JAK2, LSP1, MAPK10, MAPK12, MRC2, MYD88, NOD2, PLK3, SRC, TCIRG1, TGFB1, TLR1, TLR2, TLR4, TLR6, VDR |
| hsa04145 | Phagosome | 48 | 5.92E-11 | 4.51E-11 | ATP6V0A1, ATP6V0D1, ATP6V0E2, ATP6V1B2, C3, CALR, CD14, CD36, CLEC7A, CORO1A, CTSL, CTSS, CYBA, CYBB, FCAR, FCGR1A, FCGR2A, FCGR2B, FCGR3A, FCGR3B, HLA-B, HLA-C, HLA-DMB, HLA-DPA1, HLA-DPB1, HLA-DQA1, HLA-DQB1, HLA-DRA, HLA-DRB1, HLA-DRB5, HLA-E, ITGAM, ITGB2, ITGB5, MARCO, MRC2, MSR1, NCF1, NCF2, OLR1, RAB7B, SCARB1, TCIRG1, THBS1, TLR2, TLR4, TLR6, TUBA4A |
| hsa04380 | Osteoclast differentiation | 41 | 1.36E-09 | 1.04E-09 | BLNK, CSF1R, CYBA, FCGR1A, FCGR2A, FCGR2B, FCGR3A, FCGR3B, FOS, FOSL2, FYN, IFNGR1, IFNGR2, JUNB, LILRA1, LILRA2, LILRA4, LILRA5, LILRA6, LILRB1, LILRB2, LILRB3, LILRB4, MAP2K6, MAPK10, MAPK12, MITF, NCF1, NCF2, NFATC2, NFKB2, OSCAR, PPARG, SIRPA, SIRPB1, SPI1, TEC, TGFB1, TGFBR2, TNFRSF11A, TYROBP |
| hsa04142 | Lysosome | 40 | 1.31E-08 | 9.96E-09 | ABCA2, AP1S2, AP1S3, ARSA, ARSG, ASAH1, ATP6V0A1, ATP6V0D1, CD63, CLTCL1, CTSB, CTSD, CTSF, CTSH, CTSL, CTSS, CTSW, CTSZ, DMXL2, FUCA1, GAA, GGA2, GM2A, GNS, GUSB, HEXB, IGF2R, LAPTM4B, LAPTM5, LIPA, MAN2B1, NAGA, NCOA7, NPC2, PPT1, PSAP, SGSH, SLC11A1, SORT1, TCIRG1 |
| hsa05145 | Toxoplasmosis | 32 | 3.18E-06 | 2.42E-06 | ALOX5, BCL2, BIRC3, CASP3, CCR5, HLA-DMB, HLA-DPA1, HLA-DPB1, HLA-DQA1, HLA-DQB1, HLA-DRA, HLA-DRB1, HLA-DRB5, HSPA1A, HSPA6, IFNGR1, IFNGR2, IL10, IL10RA, IRGM, ITGA6, JAK2, LDLR, LY96, MAP2K6, MAPK10, MAPK12, MYD88, PIK3R5, TGFB1, TLR2, TLR4 |
| hsa05150 | Staphylococcus aureus infection | 29 | 3.18E-06 | 2.42E-06 | C2, C3, C3AR1, C5AR1, CAMP, FCAR, FCGR1A, FCGR2A, FCGR2B, FCGR3A, FCGR3B, FPR1, FPR2, HLA-DMB, HLA-DPA1, HLA-DPB1, HLA-DQA1, HLA-DQB1, HLA-DRA, HLA-DRB1, HLA-DRB5, ICAM1, IL10, ITGAL, ITGAM, ITGB2, KRT23, PTAFR, SELPLG |
| hsa04640 | Hematopoietic cell lineage | 28 | 2.14E-05 | 1.63E-05 | CD14, CD1C, CD1D, CD1E, CD36, CD37, CD4, CD55, CD59, CR1, CSF1R, CSF2RA, FCER2, FCGR1A, HLA-DMB, HLA-DPA1, HLA-DPB1, HLA-DQA1, HLA-DQB1, HLA-DRA, HLA-DRB1, HLA-DRB5, IL1R2, IL4R, IL6R, ITGA4, ITGA6, ITGAM |
| hsa04514 | Cell adhesion molecules | 37 | 4.66E-05 | 3.54E-05 | CADM1, CD276, CD4, CD86, CD99, F11R, HLA-B, HLA-C, HLA-DMB, HLA-DPA1, HLA-DPB1, HLA-DQA1, HLA-DQB1, HLA-DRA, HLA-DRB1, HLA-DRB5, HLA-E, ICAM1, ITGA4, ITGA6, ITGAL, ITGAM, ITGB2, ITGB7, JAM3, MPZL1, NRXN2, NTNG1, NTNG2, PECAM1, PTPRC, SDC2, SDC4, SELPLG, SIGLEC1, SPN, VCAN |
| hsa05323 | Rheumatoid arthritis | 26 | 5.54E-05 | 4.22E-05 | ATP6V0A1, ATP6V0D1, ATP6V0E2, ATP6V1B2, CD86, CTSL, CXCL1, FOS, HLA-DMB, HLA-DPA1, HLA-DPB1, HLA-DQA1, HLA-DQB1, HLA-DRA, HLA-DRB1, HLA-DRB5, ICAM1, IL15, ITGAL, ITGB2, TCIRG1, TGFB1, TLR2, TLR4, TNFRSF11A, TNFSF13B |
| hsa05134 | Legionellosis | 19 | 7.21E-05 | 5.49E-05 | APAF1, C3, CASP1, CASP3, CD14, CR1, CXCL1, HSPA1A, HSPA6, ITGAM, ITGB2, MYD88, NAIP, NFKB2, NLRC4, PYCARD, TLR2, TLR4, TLR5 |
| hsa05417 | Lipid and atherosclerosis | 45 | 8.22E-05 | 6.26E-05 | ABCG1, APAF1, BCL2, CAMK2D, CASP1, CASP3, CASP6, CD14, CD36, CXCL1, CYBA, CYBB, ERN1, FOS, HSPA1A, HSPA6, ICAM1, IRF7, JAK2, LDLR, LY96, LYN, MAP2K6, MAPK10, MAPK12, MYD88, NCF1, NCF2, NFATC2, NLRP3, OLR1, POU2F2, PPARG, PRKCA, PYCARD, RXRA, SOD2, SRC, TLR2, TLR4, TLR6, TNFRSF10B, TNFSF10, VAV2, XBP1 |
| hsa05169 | Epstein-Barr virus infection | 43 | 8.22E-05 | 6.26E-05 | APAF1, BCL2, BCL2L11, BLNK, CALR, CASP3, CCND3, CDK6, CDKN1A, CXCL10, ENTPD1, FCER2, GADD45B, HLA-B, HLA-C, HLA-DMB, HLA-DPA1, HLA-DPB1, HLA-DQA1, HLA-DQB1, HLA-DRA, HLA-DRB1, HLA-DRB5, HLA-E, ICAM1, IRF7, ITGAL, LYN, MAP2K6, MAPK10, MAPK12, MDM2, MYC, MYD88, NEDD4, NFKB2, OAS1, OAS2, RB1, SAP30, TLR2, TNFAIP3, VIM |
| hsa04659 | Th17 cell differentiation | 28 | 8.22E-05 | 6.26E-05 | AHR, CD4, FOS, HLA-DMB, HLA-DPA1, HLA-DPB1, HLA-DQA1, HLA-DQB1, HLA-DRA, HLA-DRB1, HLA-DRB5, IFNGR1, IFNGR2, IL12RB1, IL2RG, IL4R, IL6R, IL6ST, JAK2, MAPK10, MAPK12, NFATC2, PRKCQ, RARA, RUNX1, RXRA, TGFB1, TGFBR2 |
| hsa04625 | C-type lectin receptor signaling pathway | 26 | 0.000344 | 0.000262 | BCL3, CARD9, CASP1, CLEC1B, CLEC4D, CLEC4E, CLEC7A, EGR2, FCER1G, IL10, ITPR2, LSP1, MAPK10, MAPK12, MDM2, MRAS, NFATC2, NFKB2, NLRP3, PAK1, PLK3, PRKCD, PTGS2, PYCARD, RRAS, SRC |
| hsa04672 | Intestinal immune network for IgA production | 16 | 0.000402 | 0.000306 | CD86, CXCR4, HLA-DMB, HLA-DPA1, HLA-DPB1, HLA-DQA1, HLA-DQB1, HLA-DRA, HLA-DRB1, HLA-DRB5, IL10, IL15, ITGA4, ITGB7, TGFB1, TNFSF13B |
| hsa05321 | Inflammatory bowel disease | 19 | 0.000402 | 0.000306 | HLA-DMB, HLA-DPA1, HLA-DPB1, HLA-DQA1, HLA-DQB1, HLA-DRA, HLA-DRB1, HLA-DRB5, IFNGR1, IFNGR2, IL10, IL12RB1, IL2RG, IL4R, NOD2, TGFB1, TLR2, TLR4, TLR5 |
| hsa05416 | Viral myocarditis | 18 | 0.000422 | 0.000321 | CASP3, CD55, CD86, FYN, HLA-B, HLA-C, HLA-DMB, HLA-DPA1, HLA-DPB1, HLA-DQA1, HLA-DQB1, HLA-DRA, HLA-DRB1, HLA-DRB5, HLA-E, ICAM1, ITGAL, ITGB2 |
| hsa04670 | Leukocyte transendothelial migration | 27 | 0.000536 | 0.000408 | ARHGAP5, CD99, CTNNA1, CXCR4, CYBA, CYBB, F11R, ICAM1, ITGA4, ITGAL, ITGAM, ITGB2, JAM3, MAPK12, MMP2, NCF1, NCF2, PECAM1, PRKCA, PRKCB, RASSF5, RHOH, SIPA1, TXK, VASP, VAV2, VCL |
| hsa05202 | Transcriptional misregulation in cancer | 39 | 0.000545 | 0.000415 | BCL2A1, BCL6, BIRC3, CD14, CD86, CDK14, CDKN1A, CEBPB, CSF1R, DUSP6, ERG, ETV6, FCGR1A, GADD45B, ID2, IGF1R, IL1R2, ITGAM, ITGB7, KMT2A, LDB1, MDM2, MITF, MYC, MYCN, NFKBIZ, PLAU, PPARG, RARA, RUNX1, RXRA, SLC45A3, SMAD1, SPI1, SPINT1, TGFBR2, WT1, ZBTB16, ZEB1 |
|  |  |  |  |  |  |
|  |  |  |  |  |  |
