# Supplementary material for: Immune infiltration-related genes regulate the progression of AML by invading the bone marrow microenvironment
Source: Front Immunol. 2024 Jul 12;15:1409945. doi: 10.3389/fimmu.2024.1409945 (PMC11272452; doi:10.3389/fimmu.2024.1409945)
Supplement: Supplementary file 12 [file Table_4.docx]

| Table S4. GO and KEGG enrichment analysis of 178 DEGs | | | | | |
| --- | --- | --- | --- | --- | --- |
| **ID** | **Description** | **Count** | **p-value** | **FDR** | **mRNAs** |
| **BP terms** |  |  |  |  |  |
| GO:0050900 | leukocyte migration | 55 | 2.45E-12 | 2.01E-12 | ADA, BCR, BST1, C5AR1, C5AR2, CALR, CCL23, CCR1, CCR2, CCR5, CD200R1, CD99, CMKLR1, CX3CR1, CXCL10, CXCL16, CXCR1, CXCR2, FCER1G, FFAR2, FPR2, GCSAML, HCK, HMOX1, IL10, IL17RA, ITGA4, ITGA6, ITGA7, ITGB2, JAM3, LEP, LGALS3, MDK, NINJ1, NLRP12, NOD2, PADI2, PLA2G7, PTAFR, PTPRO, RHOH, S100A12, S100A8, S100A9, SERPINE1, SPN, SPNS2, ST3GAL4, THBS1, TNFAIP6, TNFRSF11A, TREM1, TRPM4, YES1 |
| GO:0001819 | positive regulation of cytokine production | 61 | 5.47E-12 | 4.48E-12 | ADRA2A, AIM2, APOA2, C5AR1, CADM1, CCR2, CD14, CD4, CD86, CLEC7A, CYBB, EPHB2, F2R, FCER1G, FCN1, FFAR2, FGR, HHLA2, HMOX1, IL10, IL17RA, IRF8, LEP, LILRA2, LILRA5, LILRB1, LILRB2, LRRK2, LY96, MDK, MEFV, MMP8, MNDA, NFAM1, NLRC4, NLRP12, NOD2, OAS1, OSM, POU2F2, PRKCQ, PTAFR, PTPRJ, RAB7B, SCIMP, SERPINE1, SLC11A1, SLC7A5, SPN, SPTBN1, SULF2, THBS1, TLR4, TLR5, TLR7, TLR8, TNFRSF8, TRIM6, TXK, ZBTB20, ZFPM1 |
| GO:0002683 | negative regulation of immune system process | 52 | 2.67E-09 | 2.19E-09 | ADA, APOA2, BCL6, BCR, BPI, C5AR2, CASP3, CCR2, CD200R1, CD59, CD86, CD96, CDK6, CR1, CX3CR1, FCGR2B, FGL2, FGR, FSTL3, GATA2, GRN, HCK, HFE, HMOX1, IL10, IL31RA, LGALS3, LILRB1, LILRB2, LILRB3, LILRB4, MAFB, MDK, MNDA, MYC, NLRC3, NOD2, OAS1, PADI2, PTPRJ, SAMHD1, SLA2, SPN, THBS1, TLR4, TMEM176A, TMEM176B, TNFAIP6, VSIG4, YES1, ZBTB46, ZFPM1 |
| GO:0030595 | leukocyte chemotaxis | 37 | 2.67E-09 | 2.19E-09 | BST1, C5AR1, C5AR2, CALR, CCL23, CCR1, CCR2, CCR5, CMKLR1, CX3CR1, CXCL10, CXCL16, CXCR1, CXCR2, FCER1G, FFAR2, FPR2, IL10, IL17RA, ITGB2, JAM3, LGALS3, MDK, NINJ1, NOD2, PADI2, PLA2G7, PTPRO, S100A12, S100A8, S100A9, SERPINE1, THBS1, TNFAIP6, TNFRSF11A, TREM1, TRPM4 |
| GO:0006909 | phagocytosis | 42 | 9.82E-09 | 8.04E-09 | APOA2, BCR, C2, CALR, CCR2, CD14, CD93, CLEC7A, FCER1G, FCGR1A, FCGR2B, FCN1, FGR, FPR2, GATA2, HCK, IGLL1, IL2RG, IRF8, ITGAM, ITGB2, LEP, LRP1, MARCO, MSR1, MYO7A, NCF2, NOD2, P2RY6, PLA2G6, PLD2, PTPRJ, RAB31, RAB39A, RAB7B, RHOBTB1, RHOH, SIRPB1, SLC11A1, THBS1, TLR4, YES1 |
| GO:0002697 | regulation of immune effector process | 45 | 1.22E-08 | 1.00E-08 | APOA2, BCL6, BCR, CADM1, CCR2, CD1C, CD1D, CD1E, CD59, CD86, CD96, CLEC7A, CR1, CX3CR1, EPHB2, FCER2, FCGR2B, FCN1, FFAR2, FGL2, FGR, GATA2, GRN, HFE, HMOX1, IL10, ITGAM, ITGB2, LEP, LGALS3, LILRB1, LILRB4, MYB, NCF1, NOD2, PTAFR, PTPRJ, SCIMP, SLC7A5, TLR4, TNFRSF1B, TNFRSF4, TRIM6, TRPM4, VSIG4 |
| GO:0002764 | immune response-regulating signaling pathway | 54 | 1.56E-08 | 1.28E-08 | ADA, BCL2, C5AR1, C5AR2, CD14, CD200R1, CLEC7A, CMKLR1, CR1, CTSS, FCER1G, FCGR2B, FCGR3A, FCN1, FFAR2, FGR, FPR1, FPR2, GCSAML, HCK, HHLA2, IGLL1, IRGM, LGALS3, LILRA2, LILRB1, LILRB2, LILRB4, LY96, MNDA, NFAM1, NFATC2, NINJ1, NOD2, OAS1, OASL, PLA2G6, PLD2, PRKCQ, PTPRJ, RAB7B, RNF125, SCIMP, SLA2, SLC15A3, SLC46A2, TEC, TESPA1, TLR4, TLR5, TLR7, TLR8, TXK, YES1 |
| GO:1902105 | regulation of leukocyte differentiation | 39 | 2.02E-08 | 1.65E-08 | ADA, BCL6, CCR1, CCR2, CD4, CD86, CDK6, CR1, FCGR2B, FGL2, FSTL3, GATA2, IL10, LILRB1, LILRB2, LILRB3, LILRB4, MAFB, MDK, MYB, MYC, NFAM1, RHOH, SLC46A2, SLC9B2, SOX12, SOX4, TESPA1, TLR4, TMEM176A, TMEM176B, TMEM64, TNFAIP6, TOX, ZBTB16, ZBTB46, ZEB1, ZFPM1, ZMIZ1 |
| GO:0032103 | positive regulation of response to external stimulus | 49 | 1.52E-07 | 1.25E-07 | ABCC1, AIM2, ALOX5AP, C5AR1, CADM1, CALR, CASP6, CCR1, CCR2, CD1D, CLEC7A, CMKLR1, CX3CR1, CXCL10, FCN1, FFAR2, FPR2, GRN, HCK, IGF1R, IL17RA, IRGM, LILRA2, LILRA5, LRRK2, LY86, LY96, MDK, MMP8, MNDA, NINJ1, NLRC4, NLRP12, NOD2, OASL, OSM, PLA2G7, S100A12, S100A8, S100A9, SCIMP, SERPINE1, THBS1, TLR4, TLR7, TLR8, TNFRSF11A, TRIM6, TXK |
| GO:0050867 | positive regulation of cell activation | 48 | 2.65E-07 | 2.17E-07 | ADA, BCL2, BCL6, BST1, CCR2, CD1D, CD4, CD86, CLEC7A, CR1, EPHB2, FCGR3A, FGR, GATA2, HHLA2, IGFBP2, IGLL1, IL10, ITGAM, ITGB2, LEP, LILRA2, LILRA5, LILRB1, LILRB2, LILRB4, LRRK2, MDK, MMP8, MPL, MYB, NFATC2, NOD2, PRKCQ, PTAFR, RHOH, SIRPB1, SLC7A1, SOX12, SOX4, TESPA1, THBS1, TLR4, TNFRSF4, TOX, YES1, ZBTB16, ZMIZ1 |
| GO:0007159 | leukocyte cell-cell adhesion | 44 | 2.85E-07 | 2.33E-07 | ADA, BCL6, CASP3, CCR2, CD1D, CD4, CD86, CERCAM, CR1, CX3CR1, FCGR2B, FGL2, HFE, HHLA2, IGFBP2, IL10, ITGA4, ITGB2, KLF4, LEP, LGALS3, LILRB1, LILRB2, LILRB4, MDK, MYB, NOD2, OLR1, PRKCQ, PTAFR, RHOH, S100A8, S100A9, SIRPB1, SLC7A1, SOX12, SOX4, SPN, ST3GAL4, TESPA1, VSIG4, YES1, ZBTB16, ZMIZ1 |
| GO:0071674 | mononuclear cell migration | 30 | 2.89E-07 | 2.36E-07 | C5AR1, CALR, CCL23, CCR1, CCR2, CCR5, CD200R1, CD99, CMKLR1, CX3CR1, CXCL10, CXCL16, CXCR1, CXCR2, FPR2, GCSAML, ITGA4, LGALS3, MDK, NLRP12, PADI2, PLA2G7, PTPRO, S100A12, SERPINE1, SPN, SPNS2, THBS1, TNFRSF11A, TRPM4 |
| GO:0060326 | cell chemotaxis | 39 | 3.07E-07 | 2.51E-07 | ABCC1, BST1, C5AR1, C5AR2, CALR, CCL23, CCR1, CCR2, CCR5, CMKLR1, CX3CR1, CXCL10, CXCL16, CXCR1, CXCR2, FCER1G, FFAR2, FPR2, IL10, IL17RA, ITGB2, JAM3, LGALS3, MDK, NINJ1, NOD2, PADI2, PLA2G7, PRKCQ, PTPRO, S100A12, S100A8, S100A9, SERPINE1, THBS1, TNFAIP6, TNFRSF11A, TREM1, TRPM4 |
| GO:0031349 | positive regulation of defense response | 37 | 3.07E-07 | 2.51E-07 | ABCC1, AIM2, ALOX5AP, CADM1, CASP6, CCR2, CD1D, CLEC7A, FCN1, FFAR2, FPR2, GRN, HCK, IL17RA, IRGM, LILRA2, LILRA5, LRRK2, MDK, MMP8, MNDA, NINJ1, NLRC4, NLRP12, NOD2, OSM, PLA2G7, S100A12, S100A8, S100A9, SERPINE1, TLR4, TLR7, TLR8, TNFRSF11A, TRIM6, TXK |
| GO:0097529 | myeloid leukocyte migration | 32 | 4.45E-07 | 3.65E-07 | BST1, C5AR1, C5AR2, CCL23, CCR1, CCR2, CD200R1, CD99, CMKLR1, CX3CR1, CXCL10, CXCR1, CXCR2, FCER1G, FPR2, IL17RA, ITGB2, JAM3, LGALS3, MDK, NOD2, PLA2G7, PTPRO, RHOH, S100A12, S100A8, S100A9, SERPINE1, THBS1, TNFAIP6, TNFRSF11A, TREM1 |
| GO:0002696 | positive regulation of leukocyte activation | 46 | 4.87E-07 | 3.98E-07 | ADA, BCL2, BCL6, BST1, CCR2, CD1D, CD4, CD86, CLEC7A, CR1, EPHB2, FCGR3A, FGR, GATA2, HHLA2, IGFBP2, IGLL1, IL10, ITGAM, ITGB2, LEP, LILRB1, LILRB2, LILRB4, LRRK2, MDK, MMP8, MPL, MYB, NFATC2, NOD2, PRKCQ, PTAFR, RHOH, SIRPB1, SLC7A1, SOX12, SOX4, TESPA1, THBS1, TLR4, TNFRSF4, TOX, YES1, ZBTB16, ZMIZ1 |
| GO:1903557 | positive regulation of tumor necrosis factor superfamily cytokine production | 21 | 5.96E-07 | 4.88E-07 | CCR2, CD14, CD86, CLEC7A, CYBB, EPHB2, LEP, LILRA2, LILRA5, LRRK2, LY96, MMP8, NOD2, OAS1, PTAFR, PTPRJ, SPN, THBS1, TLR4, TNFRSF8, ZBTB20 |
| GO:1903706 | regulation of hemopoiesis | 42 | 7.48E-07 | 6.12E-07 | ADA, BCL6, CCR1, CCR2, CD4, CD86, CDK6, CR1, FCGR2B, FGL2, FSTL3, GATA2, IL10, LDB1, LILRB1, LILRB2, LILRB3, LILRB4, MAFB, MDK, MPL, MYB, MYC, NFAM1, RAB7B, RHOH, SLC46A2, SLC9B2, SOX12, SOX4, TESPA1, TLR4, TMEM176A, TMEM176B, TMEM64, TNFAIP6, TOX, ZBTB16, ZBTB46, ZEB1, ZFPM1, ZMIZ1 |
| GO:0071706 | tumor necrosis factor superfamily cytokine production | 28 | 7.48E-07 | 6.12E-07 | BPI, C5AR2, CCR2, CD14, CD86, CLEC7A, CX3CR1, CYBB, EPHB2, IL10, LEP, LILRA2, LILRA5, LILRB1, LILRB4, LRRK2, LY96, MMP8, NLRC3, NOD2, OAS1, PTAFR, PTPRJ, SPN, THBS1, TLR4, TNFRSF8, ZBTB20 |
| GO:1903555 | regulation of tumor necrosis factor superfamily cytokine production | 28 | 7.48E-07 | 6.12E-07 | BPI, C5AR2, CCR2, CD14, CD86, CLEC7A, CX3CR1, CYBB, EPHB2, IL10, LEP, LILRA2, LILRA5, LILRB1, LILRB4, LRRK2, LY96, MMP8, NLRC3, NOD2, OAS1, PTAFR, PTPRJ, SPN, THBS1, TLR4, TNFRSF8, ZBTB20 |
| **CC terms** |  |  |  |  |  |
| GO:0009897 | external side of plasma membrane | 57 | 1.72E-11 | 1.47E-11 | ABCG1, ADA, ANXA5, ASGR2, CALR, CCR1, CCR2, CCR5, CD14, CD163, CD163L1, CD1C, CD1D, CD1E, CD200R1, CD4, CD59, CD69, CD86, CLEC10A, CLEC7A, CSF2RA, CX3CR1, CXCL10, CXCR1, CXCR2, FCER1G, FCER2, FCGR2B, FCGR3A, FCN1, FOLR2, HFE, HHLA2, IGLL1, IL13RA1, IL2RG, IL31RA, ITGA4, ITGA6, ITGA7, ITGAM, ITGAX, ITGB2, LILRB1, MPL, MSR1, PRLR, RTN4R, SCUBE1, SLC7A5, SPN, THBS1, TLR4, TLR8, TNFRSF11A, TNFRSF4 |
| GO:0030667 | secretory granule membrane | 44 | 1.31E-10 | 1.12E-10 | BST1, C5AR1, CD14, CD59, CD93, CEACAM3, CKAP4, CR1, CXCR1, CXCR2, CYBB, FCAR, FCER1G, FCGR2A, FCGR3B, FPR1, FPR2, GPR84, IGF2R, ITGAM, ITGAX, ITGB2, ITPR2, LGALS3, LILRB2, LILRB3, MCEMP1, NFAM1, OLR1, PLAU, PSAP, PTAFR, PTPRJ, RAB31, SERPINB10, SIGLEC14, SIGLEC9, SIRPB1, SLC11A1, SLC17A9, SLC27A2, SLCO4C1, STXBP5, TNFRSF1B |
| GO:0070820 | tertiary granule | 29 | 3.29E-09 | 2.82E-09 | CAMP, CD59, CD93, CDA, CFP, CR1, CTSS, CYBB, FCAR, FCER1G, FPR1, FPR2, GPR84, ITGAM, ITGAX, ITGB2, LGALS3, LILRB2, LYZ, MCEMP1, MMP8, OLR1, PLAU, PTAFR, QPCT, SERPINB10, SIGLEC14, SLC11A1, TNFAIP6 |
| GO:0070821 | tertiary granule membrane | 17 | 4.71E-07 | 4.03E-07 | CD59, CD93, CYBB, FCAR, FCER1G, FPR2, GPR84, ITGAM, ITGAX, ITGB2, LILRB2, MCEMP1, OLR1, PLAU, PTAFR, SIGLEC14, SLC11A1 |
| GO:0042581 | specific granule | 25 | 6.81E-07 | 5.83E-07 | BPI, BST1, CAMP, CD59, CD93, CEACAM3, CFP, CKAP4, CTSZ, CYBB, FCAR, FPR2, GPR84, ITGAM, ITGB2, LYZ, MCEMP1, MMP8, OLR1, PLAU, PTPRJ, QPCT, SLC27A2, SLCO4C1, TNFRSF1B |
| GO:0035579 | specific granule membrane | 18 | 1.74E-06 | 1.49E-06 | BST1, CD59, CD93, CEACAM3, CKAP4, CYBB, FCAR, FPR2, GPR84, ITGAM, ITGB2, MCEMP1, OLR1, PLAU, PTPRJ, SLC27A2, SLCO4C1, TNFRSF1B |
| GO:0101002 | ficolin-1-rich granule | 26 | 2.33E-06 | 1.99E-06 | CD93, CDA, COTL1, CR1, CRISPLD2, CTSS, CTSZ, FCAR, FCER1G, FCN1, FGL2, FPR1, FPR2, HK3, HSPA6, ITGAX, ITGB2, LGALS3, LILRB2, MNDA, QPCT, SERPINA1, SERPINB10, SIGLEC14, SLC11A1, TNFAIP6 |
| GO:0009925 | basal plasma membrane | 30 | 7.67E-06 | 6.57E-06 | ABCC1, ABCC3, ADRA2A, AQP9, ATP1B1, ATP7B, C5AR1, C5AR2, CADM1, CD1D, DST, EPCAM, KCNQ1, LRP1, MLC1, NAIP, NOD2, ORAI1, P2RY6, PALM, PKD2, SLC1A3, SLC2A9, SLC39A14, SLC7A1, SLC7A5, SLC7A7, SLC9B2, SLCO4C1, ST14 |
| GO:0045178 | basal part of cell | 31 | 9.66E-06 | 8.27E-06 | ABCC1, ABCC3, ADRA2A, AQP9, ATP1B1, ATP7B, C5AR1, C5AR2, CADM1, CD1D, DST, EPCAM, HFE, KCNQ1, LRP1, MLC1, NAIP, NOD2, ORAI1, P2RY6, PALM, PKD2, SLC1A3, SLC2A9, SLC39A14, SLC7A1, SLC7A5, SLC7A7, SLC9B2, SLCO4C1, ST14 |
| GO:0016323 | basolateral plasma membrane | 27 | 2.61E-05 | 2.24E-05 | ABCC1, ABCC3, ADRA2A, AQP9, ATP1B1, ATP7B, C5AR1, CADM1, CD1D, EPCAM, KCNQ1, LRP1, MLC1, NAIP, NOD2, ORAI1, P2RY6, PALM, PKD2, SLC2A9, SLC39A14, SLC7A1, SLC7A5, SLC7A7, SLC9B2, SLCO4C1, ST14 |
| GO:0101003 | ficolin-1-rich granule membrane | 13 | 3.49E-05 | 2.99E-05 | CD93, CR1, FCAR, FCER1G, FPR1, FPR2, ITGAX, ITGB2, LGALS3, LILRB2, SERPINB10, SIGLEC14, SLC11A1 |
| GO:0044853 | plasma membrane raft | 16 | 5.26E-04 | 4.50E-04 | ATP1B1, BVES, CR1, F2R, HCK, HMOX1, IGF1R, ITGAM, ITGB2, LRRK2, MLC1, MS4A4A, MYOF, ORAI1, SMO, TFPI |
| GO:0062023 | collagen-containing extracellular matrix | 37 | 5.37E-04 | 4.60E-04 | ADAMDEC1, ADAMTS2, AEBP1, ANXA5, CALR, CFP, COL23A1, COL24A1, COL2A1, CPA3, CSPG4, CTSF, CTSL, CTSS, CTSZ, DST, FCN1, FGL2, FLG, HSPG2, LGALS3, MDK, MEGF9, MMP2, MMP8, NTN1, NTNG2, PSAP, S100A8, S100A9, SDC2, SERPINA1, SERPINE1, TGFBI, THBS1, VCAN, VWA1 |
| GO:0098636 | protein complex involved in cell adhesion | 9 | 1.60E-03 | 1.37E-03 | ITGA4, ITGA6, ITGA7, ITGAM, ITGAX, ITGB2, JAM3, LGALS2, PLAU |
| GO:0045335 | phagocytic vesicle | 16 | 4.81E-03 | 4.12E-03 | CALR, CTSS, CYBB, IRGM, MPEG1, NCF1, NCF2, NOD2, RAB11FIP5, RAB31, RAB38, RAB39A, RAB7B, SCIMP, SLC11A1, TLR7 |
| GO:0030139 | endocytic vesicle | 29 | 4.81E-03 | 4.12E-03 | AMN, CALR, CD163, CD4, CTSL, CTSS, CYBB, EHD4, FCGR1A, IGF2R, IRGM, LRP1, MARCO, MPEG1, MSR1, NCF1, NCF2, NOD2, RAB11FIP5, RAB31, RAB38, RAB39A, RAB7B, RIN1, RIN2, SCIMP, SLC11A1, SMO, TLR7 |
| GO:0005775 | vacuolar lumen | 18 | 7.45E-03 | 6.38E-03 | BPI, CD1E, CSPG4, CTSF, CTSL, CTSS, EPDR1, GRN, HSPG2, IFI30, LYZ, MNDA, PADI2, PRSS57, PSAP, SDC2, SGSH, VCAN |
| GO:0043025 | neuronal cell body | 36 | 8.84E-03 | 7.58E-03 | ADRA2A, ALS2, AMIGO1, AVPR1B, C9orf72, CACNA1C, CASP3, CCR2, CX3CR1, CYBB, EPHB2, FEZ1, FKBP4, GRIK5, HIP1R, IGF1R, ITGA4, KCNC3, KCNC4, KCNJ2, KCNQ1, LRRK2, MAP1A, MPL, NCF1, PDE9A, RTN4R, SLC1A3, SLC8A1, SORCS2, SPTBN2, SV2A, TNFRSF1B, TRPM4, UNC5A, ZNF385A |
| GO:0060205 | cytoplasmic vesicle lumen | 27 | 8.84E-03 | 7.58E-03 | ADA, BPI, CAMP, CDA, CFP, COTL1, CRISPLD2, CTSW, CTSZ, F5, FCN1, FGR, GRN, HK3, HSPA6, LYZ, MMP8, MNDA, PADI2, PRSS57, QPCT, S100A12, S100A8, S100A9, SERPINA1, SERPINE1, THBS1 |
| GO:0045121 | membrane raft | 27 | 8.84E-03 | 7.58E-03 | ATP1B1, BVES, CASP3, CD14, CD4, CR1, F2R, HCK, HMOX1, IGF1R, ITGAM, ITGB2, KCNE1, KCNQ1, LRRK2, MLC1, MS4A4A, MYOF, NFAM1, OLR1, ORAI1, RTN4R, SMO, SYNJ2, TFPI, TNFRSF1B, UNC5A |
| **MF terms** |  |  |  |  |  |
| GO:0140375 | immune receptor activity | 35 | 6.61E-14 | 5.94E-14 | C5AR1, C5AR2, CCR1, CCR2, CCR5, CD200R1, CD4, CMKLR1, CR1, CSF2RA, CX3CR1, CXCR1, CXCR2, FCER1G, FCGR1A, FCGR2B, FCGR3A, FPR1, FPR2, IL10RA, IL13RA1, IL17RA, IL17RE, IL1R2, IL2RG, IL31RA, LILRA1, LILRA2, LILRA5, LILRA6, LILRB1, LILRB2, LILRB3, MPL, PRLR |
| GO:0019865 | immunoglobulin binding | 10 | 9.72E-06 | 8.72E-06 | FCAR, FCER1G, FCER2, FCGR1A, FCGR2A, FCGR2B, FCGR3A, FCGR3B, LGALS3, LILRA2 |
| GO:0038187 | pattern recognition receptor activity | 10 | 1.62E-05 | 1.46E-05 | CD14, CLEC7A, FCN1, LY96, MARCO, NOD2, PTAFR, TLR4, TLR7, TLR8 |
| GO:0004896 | cytokine receptor activity | 18 | 2.91E-05 | 2.61E-05 | CCR1, CCR2, CCR5, CD4, CMKLR1, CSF2RA, CX3CR1, CXCR1, CXCR2, IL10RA, IL13RA1, IL17RA, IL17RE, IL1R2, IL2RG, IL31RA, MPL, PRLR |
| GO:0032396 | inhibitory MHC class I receptor activity | 7 | 2.91E-05 | 2.61E-05 | LILRA1, LILRA2, LILRA5, LILRA6, LILRB1, LILRB2, LILRB3 |
| GO:0038024 | cargo receptor activity | 15 | 1.61E-04 | 1.45E-04 | AMN, ASGR2, CD163, CD163L1, CXCL16, FOLR2, FPR2, ITGAM, ITGB2, LRP1, LRP12, LRP1B, MARCO, MSR1, OLR1 |
| GO:0001846 | opsonin binding | 7 | 2.49E-04 | 2.24E-04 | CALR, CD93, CLEC7A, CR1, ITGAM, ITGB2, VSIG4 |
| GO:0019864 | IgG binding | 6 | 2.49E-04 | 2.24E-04 | FCER1G, FCGR1A, FCGR2A, FCGR2B, FCGR3A, FCGR3B |
| GO:0032393 | MHC class I receptor activity | 7 | 3.29E-04 | 2.95E-04 | LILRA1, LILRA2, LILRA5, LILRA6, LILRB1, LILRB2, LILRB3 |
| GO:0004875 | complement receptor activity | 6 | 3.85E-04 | 3.45E-04 | C5AR1, C5AR2, CMKLR1, CR1, FPR1, FPR2 |
| GO:0019955 | cytokine binding | 19 | 7.57E-04 | 6.80E-04 | CCR1, CCR2, CCR5, CD4, CSF2RA, CX3CR1, CXCR1, CXCR2, IL10RA, IL13RA1, IL1R2, IL1RN, IL2RG, IL31RA, ITGA4, PRLR, THBS1, TNFRSF11A, TNFRSF1B |
| GO:0001848 | complement binding | 7 | 8.77E-04 | 7.88E-04 | CALR, CD59, CD93, CR1, ITGAM, ITGB2, VSIG4 |
| GO:0033218 | amide binding | 36 | 1.81E-03 | 1.62E-03 | ACOT11, AVPR1B, CALR, CD14, CD1C, CD1D, CD1E, CMKLR1, EPDR1, EPHB2, FCGR2B, FKBP4, FOLR2, FPR2, GNRHR, GSTM2, HSPG2, IGF1R, ITGAM, ITGB2, LAPTM4B, LILRB2, LRP1, MARCO, MSR1, NMUR1, NOD2, NPR2, PLTP, PRLR, PSAP, PTGDR2, RTN4R, SLC7A5, SSTR2, TLR4 |
| GO:0001968 | fibronectin binding | 8 | 2.21E-03 | 1.98E-03 | CTSL, CTSS, FSTL3, ITGA4, LILRB4, MMP2, THBS1, TNFAIP6 |
| GO:0001637 | G protein-coupled chemoattractant receptor activity | 7 | 4.45E-03 | 4.00E-03 | CCR1, CCR2, CCR5, CMKLR1, CX3CR1, CXCR1, CXCR2 |
| GO:0004950 | chemokine receptor activity | 7 | 4.45E-03 | 4.00E-03 | CCR1, CCR2, CCR5, CMKLR1, CX3CR1, CXCR1, CXCR2 |
| GO:0001653 | peptide receptor activity | 18 | 5.61E-03 | 5.03E-03 | AVPR1B, CCR1, CCR2, CCR5, CMKLR1, CX3CR1, CXCR1, CXCR2, F2R, FPR1, FPR2, GPR84, NMUR1, NPR2, RXFP2, SORCS1, SORCS2, SSTR2 |
| GO:0008528 | G protein-coupled peptide receptor activity | 17 | 1.00E-02 | 9.01E-03 | AVPR1B, CCR1, CCR2, CCR5, CMKLR1, CX3CR1, CXCR1, CXCR2, F2R, FPR1, FPR2, GPR84, NMUR1, RXFP2, SORCS1, SORCS2, SSTR2 |
| GO:0005041 | low-density lipoprotein particle receptor activity | 5 | 1.34E-02 | 1.20E-02 | CXCL16, LRP1, LRP12, LRP1B, OLR1 |
| GO:0016493 | C-C chemokine receptor activity | 6 | 1.37E-02 | 1.23E-02 | CCR1, CCR2, CCR5, CX3CR1, CXCR1, CXCR2 |
| **KEGG** |  |  |  |  |  |
| hsa04145 | Phagosome | 23 | 4.01E-04 | 3.78E-04 | CALR, CD14, CLEC7A, CTSL, CTSS, CYBB, FCAR, FCGR1A, FCGR2A, FCGR2B, FCGR3A, FCGR3B, ITGAM, ITGB2, MARCO, MRC2, MSR1, NCF1, NCF2, OLR1, RAB7B, THBS1, TLR4 |
| hsa04380 | Osteoclast differentiation | 20 | 5.95E-04 | 5.61E-04 | FCGR1A, FCGR2A, FCGR2B, FCGR3A, FCGR3B, LILRA1, LILRA2, LILRA5, LILRA6, LILRB1, LILRB2, LILRB3, LILRB4, MAPK12, NCF1, NCF2, NFATC2, SIRPB1, TEC, TNFRSF11A |
| hsa04610 | Complement and coagulation cascades | 15 | 1.17E-03 | 1.10E-03 | C2, C5AR1, CD59, CR1, F2R, F5, ITGAM, ITGAX, ITGB2, PLAU, SERPINA1, SERPINB2, SERPINE1, TFPI, VSIG4 |
| hsa05150 | Staphylococcus aureus infection | 16 | 1.17E-03 | 1.10E-03 | C2, C5AR1, CAMP, FCAR, FCGR1A, FCGR2A, FCGR2B, FCGR3A, FCGR3B, FPR1, FPR2, IL10, ITGAM, ITGB2, KRT23, PTAFR |
| hsa05140 | Leishmaniasis | 14 | 1.17E-03 | 1.10E-03 | CR1, CYBB, FCGR1A, FCGR2A, FCGR3A, FCGR3B, IL10, ITGA4, ITGAM, ITGB2, MAPK12, NCF1, NCF2, TLR4 |
| hsa05152 | Tuberculosis | 23 | 1.20E-03 | 1.13E-03 | BCL2, CAMP, CASP3, CD14, CLEC7A, CR1, CTSS, FCER1G, FCGR1A, FCGR2A, FCGR2B, FCGR3A, FCGR3B, IL10, IL10RA, ITGAM, ITGAX, ITGB2, MAPK12, MRC2, NOD2, TLR4, VDR |
| hsa04060 | Cytokine-cytokine receptor interaction | 31 | 2.21E-03 | 2.08E-03 | CCL23, CCR1, CCR2, CCR5, CD4, CSF2RA, CX3CR1, CXCL10, CXCL16, CXCR1, CXCR2, GDF11, IL10, IL10RA, IL13RA1, IL17RA, IL17RE, IL1R2, IL1RN, IL2RG, IL31RA, LEP, MPL, OSM, PRLR, TNFRSF11A, TNFRSF1B, TNFRSF4, TNFRSF8, TNFSF10, TNFSF12 |
| hsa04640 | Hematopoietic cell lineage | 14 | 1.22E-02 | 1.15E-02 | CD14, CD1C, CD1D, CD1E, CD4, CD59, CR1, CSF2RA, FCER2, FCGR1A, IL1R2, ITGA4, ITGA6, ITGAM |
| hsa05134 | Legionellosis | 10 | 1.22E-02 | 1.15E-02 | CASP3, CD14, CR1, HSPA6, ITGAM, ITGB2, NAIP, NLRC4, TLR4, TLR5 |
| hsa04613 | Neutrophil extracellular trap formation | 21 | 1.22E-02 | 1.15E-02 | AQP9, C5AR1, CAMP, CLEC7A, CR1, CYBB, FCGR1A, FCGR2A, FCGR3A, FCGR3B, FPR1, FPR2, ITGAM, ITGB2, MAPK12, NCF1, NCF2, SIGLEC9, TLR4, TLR7, TLR8 |
| hsa05146 | Amoebiasis | 14 | 1.22E-02 | 1.15E-02 | CASP3, CD14, CD1C, CD1D, CD1E, GNA15, IL10, IL1R2, ITGAM, ITGB2, PRKACB, RAB7B, SERPINB10, TLR4 |
| hsa04061 | Viral protein interaction with cytokine and cytokine receptor | 13 | 2.96E-02 | 2.79E-02 | CCL23, CCR1, CCR2, CCR5, CX3CR1, CXCL10, CXCR1, CXCR2, IL10, IL10RA, IL2RG, TNFRSF1B, TNFSF10 |
| hsa04142 | Lysosome | 15 | 4.75E-02 | 4.48E-02 | ABCA2, AP1S2, AP1S3, CTSF, CTSL, CTSS, CTSW, CTSZ, IGF2R, LAPTM4B, NCOA7, PSAP, SGSH, SLC11A1, SORT1 |
| hsa05168 | Herpes simplex virus 1 infection | 39 | 5.02E-02 | 4.74E-02 | BCL2, CALR, CASP3, CFP, OAS1, PILRA, POU2F2, ZNF107, ZNF135, ZNF154, ZNF175, ZNF253, ZNF254, ZNF256, ZNF257, ZNF273, ZNF285, ZNF320, ZNF347, ZNF382, ZNF415, ZNF429, ZNF43, ZNF439, ZNF460, ZNF471, ZNF486, ZNF506, ZNF528, ZNF544, ZNF667, ZNF677, ZNF717, ZNF730, ZNF737, ZNF772, ZNF792, ZNF793, ZNF91 |
| hsa04670 | Leukocyte transendothelial migration | 13 | 7.25E-02 | 6.83E-02 | ARHGAP5, CD99, CYBB, ITGA4, ITGAM, ITGB2, JAM3, MAPK12, MMP2, NCF1, NCF2, RHOH, TXK |
| hsa05133 | Pertussis | 10 | 7.25E-02 | 6.83E-02 | C2, CASP3, CD14, IL10, IRF8, ITGAM, ITGB2, LY96, MAPK12, TLR4 |
| hsa04514 | Cell adhesion molecules | 16 | 7.91E-02 | 7.46E-02 | CADM1, CD4, CD86, CD99, ITGA4, ITGA6, ITGAM, ITGB2, JAM3, MPZL1, NRXN2, NTNG2, SDC2, SIGLEC1, SPN, VCAN |
