# Supplementary material for: Immune infiltration-related genes regulate the progression of AML by invading the bone marrow microenvironment
Source: Front Immunol. 2024 Jul 12;15:1409945. doi: 10.3389/fimmu.2024.1409945 (PMC11272452; doi:10.3389/fimmu.2024.1409945)
Supplement: Supplementary file 13 [file Table_5.docx]

Table S5. GSEA analysis between two risk groups

| **Description** | **setSize** | **enrichmentScore** | **NES** | **p.adjust** | **FDR** | **rank** | **core_enrichment** |
| --- | --- | --- | --- | --- | --- | --- | --- |
| REACTOME_NEUTROPHIL_DEGRANULATION | 65 | 0.60 | 3.59 | 6.35E-09 | 4.68E-09 | 341 | CD14, HK3, SIGLEC9, S100A9, C5AR1, S100A8, S100A12, FPR1, SLC11A1, LYZ, SIRPB1, FGL2, FCN1, LILRB2, FPR2, CDA, CRISPLD2, FGR, CYBB, LILRB3, CTSS, CAMP, TNFRSF1B, PTAFR, LGALS3, SERPINA1, FCAR, MCEMP1, MMP8, FCGR3B, FCER1G, CR1, ITGAM, PADI2, CD93, HSPA6, CKAP4, CXCR1, MNDA, QPCT, FCGR2A, NFAM1, SLCO4C1, CXCR2, SIGLEC14, PTPRJ, BST1, RAB31, CFP, GPR84, IGF2R, BPI, ITGAX, GRN, OLR1, CEACAM3, CTSZ, PSAP, COTL1, ITGB2, TNFAIP6, SERPINB10 |
| REACTOME_INNATE_IMMUNE_SYSTEM | 110 | 0.52 | 3.51 | 6.35E-09 | 4.68E-09 | 342 | CD300E, TLR8, CD14, HK3, MEFV, SIGLEC9, S100A9, C5AR1, NCF1, S100A8, CLEC10A, S100A12, TLR5, CLEC7A, FPR1, SLC11A1, LYZ, SIRPB1, FGL2, CD300LB, TLR7, FCN1, CCR2, LILRB2, FPR2, C2, CDA, CRISPLD2, FGR, CYBB, HMOX1, NCF2, LILRB3, CTSS, FCGR3A, CAMP, TNFRSF1B, RNASE6, PTAFR, LY96, LGALS3, SERPINA1, FCAR, NLRC4, TLR4, MCEMP1, MMP8, FCGR3B, FCER1G, CR1, ITGAM, CD4, PADI2, TREM1, DTX4, CD93, HSPA6, CKAP4, CXCR1, LY86, MNDA, QPCT, PLD2, FCGR2A, CTSL, NFAM1, NOD2, SLCO4C1, CXCR2, HCK, SIGLEC14, C5AR2, PTPRJ, BST1, RAB31, CFP, GPR84, FCGR1A, IGF2R, BPI, ITGAX, AIM2, GRN, OLR1, CEACAM3, CTSZ, PSAP, COTL1, ITGB2, TNFAIP6, SERPINB10, RNF125 |
| REACTOME_INTERLEUKIN_10_SIGNALING | 13 | 0.72 | 2.59 | 4.74E-04 | 3.49E-04 | 244 | IL1R2, IL10, FPR1, CCR1, CCR2, IL1RN, CD86, CCR5, TNFRSF1B, PTAFR, CXCL10, FCER2, IL10RA |
| REACTOME_ADAPTIVE_IMMUNE_SYSTEM | 61 | 0.39 | 2.32 | 2.22E-03 | 1.64E-03 | 164 | CD300E, CD14, SIGLEC9, S100A9, LILRB4, SIGLEC1, CD1D, LILRA5, LILRA6, NCF1, S100A8, SIGLEC7, CD300LB, IFI30, LILRB2, CD86, LILRB1, CD300C, LILRA1, CYBB, NCF2, LILRB3, CD101, CTSS, FCGR3A, LY96, TLR4 |
| REACTOME_TOLL_LIKE_RECEPTOR_CASCADES | 17 | 0.59 | 2.32 | 3.56E-03 | 2.63E-03 | 258 | TLR8, CD14, S100A9, S100A8, S100A12, TLR5, TLR7, CTSS, LY96, TLR4, ITGAM, LY86, CTSL, NOD2 |
| REACTOME_IMMUNOREGULATORY_INTERACTIONS_BETWEEN_A_LYMPHOID_AND_A_NON_LYMPHOID_CELL | 28 | 0.48 | 2.26 | 4.98E-03 | 3.67E-03 | 142 | CD300E, SIGLEC9, LILRB4, SIGLEC1, CD1D, LILRA5, LILRA6, SIGLEC7, CD300LB, LILRB2, LILRB1, CD300C, LILRA1, LILRB3, FCGR3A |
| KEGG_CHEMOKINE_SIGNALING_PATHWAY | 14 | 0.59 | 2.19 | 1.25E-02 | 9.21E-03 | 272 | NCF1, CCR1, CX3CR1, CCR2, CCL23, FGR, CCR5, CXCL16, CXCL10, CXCR1, CXCR2, HCK |
| REACTOME_CYTOKINE_SIGNALING_IN_IMMUNE_SYSTEM | 55 | 0.35 | 2.03 | 2.56E-02 | 1.89E-02 | 312 | IL31RA, PRLR, IL1R2, IL10, S100A12, SERPINB2, FPR1, CCR1, IFI30, CCR2, IL1RN, CD86, CCR5, HMOX1, TNFRSF1B, SAMHD1, PTAFR, CXCL10, ITGAM, IL13RA1, CD4, FCER2, BCL6, TNFSF12, IRF8, IL10RA, OASL, NOD2, TNFRSF11A, TNFRSF8, HCK, OAS1, PTPRJ, FCGR1A, IL17RA, RHOU, ITGAX |
| REACTOME_ANTIGEN_PROCESSING_CROSS_PRESENTATION | 13 | 0.59 | 2.13 | 2.90E-02 | 2.14E-02 | 164 | CD14, S100A9, NCF1, S100A8, CYBB, NCF2, CTSS, LY96, TLR4 |
| WP_VITAMIN_D_RECEPTOR_PATHWAY | 22 | 0.48 | 2.05 | 3.05E-02 | 2.25E-02 | 247 | CD14, S100A9, S100A8, SLC8A1, LRRC25, KLF4, SLC37A2, CAMP, VDR, CDKN2B, ITGAM, TREM1, BCL6, IRF8, STS |
| WP_TYROBP_CAUSAL_NETWORK_IN_MICROGLIA | 15 | 0.54 | 2.05 | 3.05E-02 | 2.25E-02 | 202 | SLC7A7, RBM47, ADAP2, CXCL16, NCF2, TNFRSF1B, RNASE6, ITGAM, IGSF6, IL13RA1, CD4 |
| WP_MICROGLIA_PATHOGEN_PHAGOCYTOSIS_PATHWAY | 10 | 0.60 | 1.98 | 3.05E-02 | 2.25E-02 | 337 | NCF1, SIGLEC7, CYBB, NCF2, FCER1G, ITGAM, TREM1, HCK, FCGR1A, ITGB2 |
| KEGG_CYTOKINE_CYTOKINE_RECEPTOR_INTERACTION | 26 | 0.41 | 1.88 | 4.63E-02 | 3.42E-02 | 271 | PRLR, IL1R2, IL10, CCR1, CX3CR1, CCR2, CCL23, CCR5, CXCL16, TNFRSF1B, CXCL10, IL13RA1, TNFSF10, CXCR1, TNFSF12, IL10RA, CXCR2, TNFRSF11A, TNFRSF8 |
| WP_CHEMOKINE_SIGNALING_PATHWAY | 11 | 0.57 | 1.96 | 4.84E-02 | 3.57E-02 | 165 | NCF1, CCR1, CX3CR1, CCR2, FGR, CXCL16, CXCL10 |
| REACTOME_SIGNALING_BY_INTERLEUKINS | 39 | 0.36 | 1.85 | 4.84E-02 | 3.57E-02 | 207 | IL31RA, IL1R2, IL10, S100A12, SERPINB2, FPR1, CCR1, CCR2, IL1RN, CD86, CCR5, HMOX1, TNFRSF1B, PTAFR, CXCL10, ITGAM, IL13RA1, CD4, FCER2 |
